# Universal Hydrogel Carrier Enhances Bone Graft Success: Preclinical and Clinical Evaluation

**DOI:** 10.1002/adhm.202403930

**Published:** 2025-01-22

**Authors:** Dax Calder, Farshad Oveissi, Simin Maleknia, Tom Huang, Bernard Koong, Terence Abrams, Andrew Oar, Wojciech Chrzanowski, Fariba Dehghani, Ali Fathi

**Affiliations:** ^1^ Sydney Pharmacy School Faculty of Medicine and Health University of Sydney Sydney NSW 2006 Australia; ^2^ Dental School University of Western Australia Perth WA 6009 Australia; ^3^ School of Chemical and Biomolecular Engineering The University of Sydney Sydney NSW 2006 Australia; ^4^ Tetratherix Technology Pty Ltd Sydney NSW 2000 Australia; ^5^ Envision Medical Imaging Wembley WA 6014 Australia; ^6^ Gold Coast University Hospital Southport 4215 Australia; ^7^ Department of Laboratory Medicine Division of Biomolecular and Cellular Medicine Division of Clinical Immunology Karolinska Institute Division of Biomedical Engineering Department of Materials Science and Engineering Uppsala University Uppsala 75105 Sweden; ^8^ Division of Biomedical Engineering Department of Materials Science and Engineering Uppsala University Upsala 75105 Sweden

**Keywords:** bone graft, bone healing, platform technology, thermoresponsive hydrogel, universal carrier

## Abstract

Orthopedic, maxillofacial, and complex dentoalveolar bone grafting procedures that require donor‐site bone harvesting can be associated with post‐surgical complications. There has been widespread adoption of exogenously sourced particulate bone graft materials (BGM) for bone regenerative procedures; however, the particulate nature of these materials may lead to compromised healing outcomes, mainly attributed to structural collapse of the BGM, prolonged tissue healing. In this study, a fully synthetic thermoresponsive hydrogel‐based universal carrier matrix (TX) that forms flowable and shapable putties with different BGMs while spatially preserving the particles in a 3D scaffold at the implantation site is introduced. The potential synergistic effect of the carrier is investigated in combination with particulate demineralized bone matrix (DBM) in a standard muscle pouch nude mice model (n = 24) as well as in a rabbit femoral critical‐sized cortico‐cancellous bone defect model (n = 9). Finally, the clinical usability, safety, and efficacy of the carrier for the delivery of deproteinized bovine bone mineral (DBBM) are evaluated in a controlled clinical trial for extraction socket alveolar ridge preservation (ARP) (n = 11 participants). Overall, the TX carrier improved the delivery of different types of BGMs, maintaining these spatially at the implantation site with minimal inflammatory responses, resulting in favorable bone regenerative outcomes.

## Graphical abstract

TX hydrogel improves the delivery of different types of particulate bone grafts, maintaining them at the implantation sites, and resulting in favorable bone regenerative outcomes.

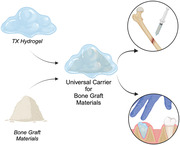

## 1 Introduction

For patients undergoing complex bone regenerative procedures to replace damaged trabecular or cortical structures, autogenous bone harvested from donor sites such as the iliac crest, rib, mandible, or calvaria is generally considered the gold standard bone graft material (BGM).^[^ ^1^ ^]^ Unfortunately, donor site harvesting requires an additional surgical procedure which can result in complications such as prolonged operation time, higher risk of infection, donor site injury, graft ischemia, and scarring.^[^ ^2^ ^]^ In contrast, the use of exogenously sourced BGM facilitates minimally invasive surgical procedures in an ambulatory outpatient setting with an inherent reduction in surgical complexity, while simultaneously avoiding the morbidity that can be associated with donor‐site bone harvesting.^[^ ^3^ ^]^ In the United States alone, a total of 1914905 orthopedic procedures were performed at ambulatory surgery centers from 2012 to 2017, with an 8.8% increase in annual procedure volume and a compound annual growth rate of 1.8%.^[^ ^4^ ^]^ Subsequently, there has been an emerging interest in the simplification of bone grafting operations that require direct BGM insertion.^[^ ^5^ ^]^

Exogenously sourced BGMs are often chosen according to certain biomechanical characteristics such as favorable mechanical stability, the ability to support angiogenesis, cellular infiltration, osteoconduction, and ideally osteoinduction.^[^ ^6^ ^]^ Exogenously sourced BGM can be classified as either synthetic materials, allografts, or xenografts.^[^ ^7^ ^]^ Synthetic bone substitutes are often based on calcium phosphate cements and calcium phosphate ceramics.^[^ ^8^ ^]^ The main drawbacks of synthetic bone substitutes are their brittleness, unpredictable resorption profile, and porosity.^[^ ^3^ ^]^ which have resulted in a more widespread adoption of allogenic and xenografts alternatives. Allogenic BGM can be provided as off‐the‐shelf products that are prepared by removing the non‐mineralized protein components or by removing the mineralized components and can be provided in either solid form such as blocks or morselized with various particle dimensions.^[^ ^6^ ^]^ For example, demineralized bone matrix (DBM) is prepared by removing all calcified mineral content resulting in a BGM that contains primarily collagenous and non‐collagenous matrix proteins Owing to the concern about zoonotic disease transmission, with respect to prion‐related disease, xenograft BGMs are intensely processed to form deproteinized bone minerals.^[^ ^9^ ^]^ Sources of commercially available xenograft BGMs include bovine (Geistlich Bio‐Oss, Geistlich Pharma AG, Wolhusen, Switzerland),^[^ ^10^ ^]^ porcine (e.g., MinerOss‐XP, Zimmer Biomet, Warsaw, Indiana, The United States of America)^[^ ^11^ ^]^ and coral exoskeleton (BioCoral, Biomatlante, Vannes, France).^[^ ^12^ ^]^

The granulated nature of particulate BGMs causes challenges including inhomogeneous administration and potential clinical complications, mostly attributed to graft migration.^[^ ^13^, ^14^, ^15^ ^]^ Hence, there has been a focus on developing carrier systems to improve the clinical effectiveness of different particulate BGMs.^[^ ^16^, ^17^, ^18^ ^]^ Examples of the particulate DBM carrier systems include natural and synthetic polymers, such as sodium hyaluronate (e.g., DBX Putty),^[^ ^19^ ^]^ glycerol (e.g., Grafton Putty),^[^ ^20^ ^]^ acellular matrix (e.g., AlloGraft™ DBM),^[^ ^21^ ^]^ lecithin (e.g., InterGro™ Putty),^[^ ^22^ ^]^ chitosan,^[^ ^23^ ^]^ sodium alginate,^[^ ^24^ ^]^ carboxymethylcellulose,^[^ ^25^ ^]^ calcium sulfate,^[^ ^26^ ^]^ polyorthoester,^[^ ^27^ ^]^ poly(lactic‐*co*‐glycolic acid),^[^ ^28^ ^]^ and poloxamer (e.g., Integra/IsoTis Accell Connexus).^[^ ^18^ ^]^ In contrast to the extensive variety of DBM carrier systems described in the literature, deproteinized bovine bone mineral (DBBM) carriers were limited to either porous sponge‐like collagen‐based carriers, i.e., Bio‐Oss Collagen (with 10% porcine collagen) which has been used extensively in ARP procedures^[^ ^29^ ^]^ or viscous carriers such as enamel matrix protein derivatives (e.g., Emdogain).^[^ ^30^ ^]^ Ideally, the carrier system should have intra‐procedure interchangeability, for example where the same system can be used with DBBM and/or DBM in a single surgical procedure according to the individual site‐specific requirements as they relate to trabecular and/or cortical bone regeneration. Most commercially available systems do not universally meet this criterion.

We previously described a hydrogel‐based platform technology (TX) for tissue‐agnostic regeneration.^[^ ^31^ ^]^ The engineered hydrogel was biocompatible, had minimal acute and systematic toxicity, and stabilized blood clots to adhere to both soft and hard tissues– supporting natural regeneration.^[^ ^31^, ^32^ ^]^ The current study aims to introduce TX as a fully synthetic hydrogel‐based universal carrier system, designed to be combined with a wide range of particulate BGMs. The primary intended mechanism of action for this hydrogel system is to improve the clinical handling of different BGMs and to spatially maintain these particulates at the implantation site to improve bone regenerative outcomes. The animal and clinical models chosen in this study include a nude mouse hind limb osteoinductive model, a rabbit femoral critical size cortico‐cancellous defect model, and a randomized controlled clinical trial pilot study involving trabecular bone regeneration of tooth extraction sockets. It is hypothesized that TX provides a hydrogel matrix that can retain particulate BGM within a 3D scaffold at the intended site, resulting in minimal cellular inflammatory activity and favorable bone regenerative outcomes, including osteoinduction, cortical bridging, and trabecular regeneration. By deliberate design, particulate DBM and DBBM are chosen in this study given that these BGMs cover a range of clinical indications in which these two families of BGMs are widely implanted.

## 2 Results and Discussion

### 2.1 Osteoinductive Characteristics of the System

**Figure 1:**
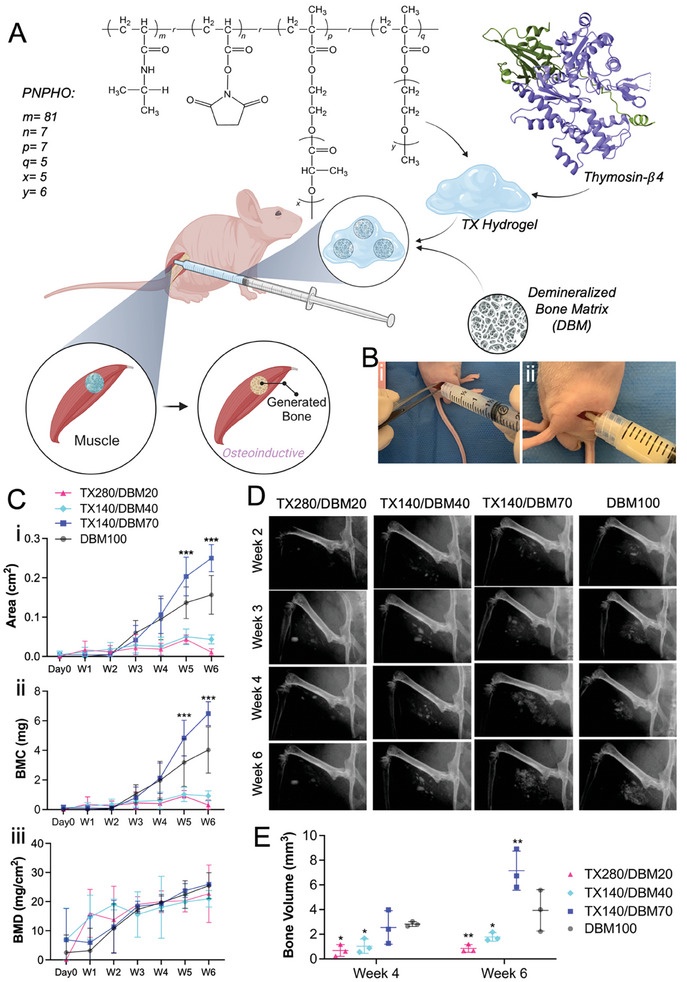
Osteoinductive study on nude mice. A) TX hydrogel formation and delivery of DBM to the limb of the nude mouse and demonstrating osteoconductivity of TX or osteoinductivity of the composite by subsequent formation of bone tissue in the limb. B) Administration of i) TX280/DBM 20 and ii) TX140/DBM70. C) DEXA measurements; i) bone area, ii) bone matrix content, and (iii) bone matrix density formed with different configurations of TX and DBM, D) Representative radiographic images of ectopic bone formation by induction of different test TX/DBM composites. E) Bone Volume measured by micro‐CT analysis. Data in all plots are expressed as mean ± SD. One‐way ANOVA followed by the Tukey test (as post‐HoC analysis) was conducted to identify the significant difference between means, statistical significance was then demonstrated as *P < 0.05, **P < 0.01, ***P < 0.001 compared with DBM100 in each time point.

The carrier hydrogel (TX) in this study consists of a synthetic polymer, namely PNPHO, crosslinked with a synthetic peptide, Thymosin‐ß4.^[^ ^33^ ^]^ PNPHO is made from random polymerization of *N*‐isopropylacrylamide (NIPAAm), polylactide‐2‐hydroxyethyl methacrylate (PLA‐HEMA), Oligo (ethylene glycol) monomethyl ether methacrylate (OEGMA), and *N*‐acryloxysuccinimide (**Figure** **1**A). The role and ratio of each component in TX hydrogel and the rationale behind the selected ratios were discussed in detail in our previous work.^[^ ^31^ ^]^ The inner ratio of the hydrogel constituents significantly affects other attributes of the hydrogel, such as stability, degradation, injectability, and mechanical properties. Such parameters were optimized in our previous work and TX140 and TX280 were shortlisted for the current application.^[^ ^31^, ^33^ ^]^ In a physiologically simulated condition (e.g., 0.5 ml sample kept in 50 ml PBS in the incubator at 37 °C), TX140 and TX280 were stable without any significant weight change for more than 2 and 10 weeks, respectively. In the first instance to investigate the potential usability of TX as a carrier system for bone grafts, a standard hind limb osteoinduction model was used.^[^ ^34^, ^35^ ^]^ By using this model, the cell‐permeable nature of the TX hydrogel system to retain graft particles in a 3D structure was studied.

TX hydrogel was mixed with osteoinductive human‐derived, demineralized bone matrix (DBM) particles at room temperature in situ prior to the animal study (Figure 1A). To confirm the modularity of TX and its potential to mix with different amounts of DBM, the graft content was varied in the range of 20 vol% to 75 vol%. Using such a wide range allowed studying the potential of the carrier to address requirements in more cost‐sensitive applications with low DBM content, e.g., dental, or for more efficacy‐driven uses, for instance in spinal fusion applications. Based on our previous studies, we initially intended to form all composite configurations with TX140 formulation. As such, composites were successfully formed with high bone graft content (between 40 vol% to 75 vol%). The excess amount of water in TX140 caused handling challenges to form formulations with low DBM content, and therefore TX280 was used to mix with 20 vol% DBM. As such, three composite configurations, namely TX280/DBM20, TX140/DBM40, TX140/DBM70 were used, and their performance was compared with DBM100 (+Control). The water intake of the prepared samples, TX280/DBM20, TX140/DBM40, TX140/DBM70, and DBM100 were 1.80 ± 0.10, 1.75 ± 0.30, 1.45 ± 0.10, and 1.10 ± 0.15 g per g of dried sample, respectively. All composites, except DBM100, were flowable and could be directly administered into the muscle pouch site (Figure 1B). For non‐flowable, DBM100 particles, the dry chips of DBM were initially hydrated with saline for injection and subsequently implanted using the lab micro spatula and the forceps followed by press‐fitting to ensure complete implantation of 100 µl of particles. Considering the primary mechanism of action for TX hydrogel as a carrier, examining signaling pathways or cell differentiation markers correlated to TX hydrogel alone at cellular and molecular levels is out of the scope of this work.

The area of bone tissue, bone matrix content (BMC), and bone matrix density (BMD) were measured at different time points. The area of bone tissue was found to be relatively small (≈0.01 cm^2^) in the first 2 weeks following implantation (Figure 1C‐i). In week 3, TX140/DBM70 and DBM100 stimulated more bone formation compared with other materials. The difference increased with time as week 4, demonstrated significantly higher (*P*<0.001) bone areas in TX140/DBM70 and DBM100 compared with any other groups. 6 weeks post‐implantation (at the termination), TX140/DBM70 demonstrated the greatest capability of bone formation (greater than 2 cm^2^ in area) that was significantly greater (*P*<0.001) than that of DBM100. Other groups revealed comparable small areas of newly formed bone tissues (lower than 0.5 cm^2^). As expected, in DBM100, bone formation was high as the residual calcium in DBM acted as nuclei for the deposition of calcium phosphate leading to easier calcification of the bone.^[^ ^36^, ^37^ ^]^ The overall higher area of bone formation with TX140/DBM70 compared with control (DBM100) is believed to be associated with the matrix of TX140 hydrogel that provided easier cell attachment through osteoinductive mechanisms.^[^ ^36^ ^]^

A similar pattern to the bone area was observed in bone matrix content (BMC), showing minimal bone formation in weeks 1 and 2 (Figure 1C‐ii). In week 3 and week 4, mouse hindlimbs implanted with TX140/DBM70 and DBM100 had significantly increased BMC. By week 5 and 6, TX140/DBM70 induced the most bone formation, followed by DBM100. Other groups had consistently low BMC of formed bone (less than 1 mg). Dual‐energy x‐ray absorptiometry (DEXA) measurements also demonstrated that all groups exhibited low BMD at less than 10 mg cm^−2^ on day 0 and week 1 (Figure 1C‐iii). Gradually, 2 weeks post‐operation, most groups had a BMD of ≈10–20 mg cm^−2^. The mice receiving TX280/DMB20, TX140/DBM70, and DMB100 displayed higher BMD of more than 20 mg cm^−2^ after 5 weeks post‐implantation. Subsequently, no specific trend was observed regarding the BMD of all TX/DBM configurations and the DBM100 group which can imply the relative osteogenic characteristics of all groups.

The ectopic bone formation of the TX/DBM implant was also investigated using Faxitron X‐ray. The representative radiographic images are shown in Figure 1D. In week 2, the newly formed bone is observed in the hindlimbs of TX140/DBM70. In week 3 and week 4, the newly generated bone is apparent in limbs implanted with almost all the tested groups, including low DBM content (e.g., 20%). In week 6, the newly formed bone of TX140/DBM70 and DBM100 seemed to be merged into one homogenous bone structure. Furthermore, the bone volume of the induced ectopic bone formation was determined by micro‐CT analysis using CTAn (Figure 1E). In week 4 post‐operation, TX280/DBM20 and TX140/DBM40 induced significantly (*P*<0.05) smaller volumes of ectopic bone tissues compared with DBM100 (0.7 ± 0.3 mm^3^ TX280/DBM20 and 1.0 ± 0.3 mm^3^ TX140/DBM40 compared with 2.8 ± 0.1 mm^3^ DBM100). In week 6, among all groups, TX140/DBM70 demonstrated the best ability to induce bone formation with a greater (*P*<0.01) volume of newly formed bone (7.2 ± 1.0 mm^3^ TX140/DBM70 compared with 3.9 ± 1.7 mm^3^ DBM100). These results endorsed the findings from the DEXA scans, and subsequently, it can be inferred that TX140 with the high concentration of DBM (TX140/DBM70) had the most osteoinductivity.

**Figure 2:**
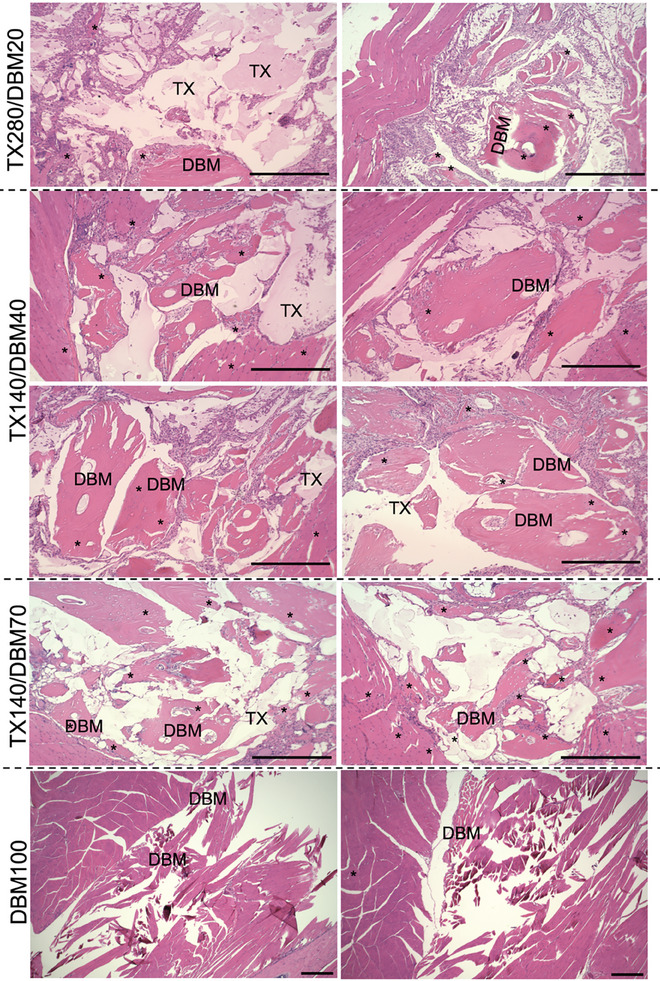
H&E stained of the implanted sites at mice hindlimbs at 6 weeks post‐implantation for different composites. TX: hydrogel remnants, DBM: Demineralized Bone Matrix. Newly regenerated ectopic bone regions are identified with *. Scales bars are 100 µm.

The representative H&E images of ectopic bone histology at 6 weeks post‐implantation are displayed in **Figure** **2**. The histological evaluations of the sites 4 weeks post‐implantation are provided and outlined in the Figures S1–S4 (Supporting Information). At 6 weeks post‐implantation time point, TX280/DBM20 induced a small amount of ectopic bone formation. The TX hydrogel could still be observed at this time point, providing evidence of the stability of TX gels for at least 6 weeks post‐implantation and its capability to retain DBM particles in place as intended. The histological assessment on TX140/DBM70 indicates a greater extent of ectopic bone formation in the sites implanted with TX140 gel encapsulating DBM, compared to the +Control (DBM100). There were still remnants of TX hydrogel throughout the area with evident cell ingrowth within the hydrogel. Similar to the results of 4 weeks, osteoblast cell layers were observed in the vicinity of DBM granules and TX hydrogels, indicating that ectopic bone formation at the site is ongoing. More importantly, TX hydrogels were entangled within the newly generated ectopic bone (asterisk, *, areas in Figure 2) where osteocytes were identified. Compared with the findings in week 4 (Figures S1–S4, Supporting Information), much less TX gel was noticed, showing the gradual resorption of the carrier system as expected. A consistent trend of significantly more ectopic bone formation with TX140/DBM70 compared with TX280/DBM20 and TX140/DBM40 was also observed.

It is worth mentioning that the +Control in this study, human DBM allograft powder (DBM100%) has been widely researched and used for bone regeneration purposes. The results suggested that the osteoinductivity of DBM is positively associated with the concentration of DBM in the hydrogel. This trend was seen in other DBM carriers, e.g., oligo(poly(ethylene glycol) fumarate at a ratio of 3:1 (DBM: Carrier) had a greater volume of formed bone than that at a ratio of 1:1.^[^ ^38^ ^]^ In the formulation with no carrier content, 100 v% DBM, the handling, and management of the particulate DBM were shown to be suboptimal during the implantation procedure.^[^ ^39^ ^]^ It was necessary to use spatulas and forceps to tuck the DBM particles into the muscle pocket, which was time‐consuming and often resulted in additional loss and waste of the DBM. In addition, the DBM powder in the muscle pouch did not seem to be homogenous and distributed evenly. Notably, the administration of the TX/DBM composite was substantially easier compared with pure DBM (Control). All TX140/DBM putties were manageable and were easily flowable and could be set well into muscle pouches. The consistent volume of the composites (100 µL) was easily implanted into the muscle pouch. All tested configurations, even with a low DBM content of 20% induced ectopic bone formation. The extent of ectopic bone formation was more evident in configurations with 40% DBM and 70% DBM contents, confirming that the use of the TX carrier system does not decrease the activity of the osteoinductive particles.

TX140/DBM70 demonstrated the greatest potential in bone induction formation in comparison to other groups, even greater than that achieved with 100% DBM content. Conversely, in the previous report on the same animal model with a thermo‐gelling DBM carrier (chitosan‐based hydrogel), the bone induction formation was higher in DBM (control) than that with the carrier,^[^ ^40^ ^]^ indicating the “synergistic” effect of TX hydrogel carrier system in combination with DBM. This may suggest that the TX carrier system at these concentrations has favorable degradation profiles, potentially allowing the slow release of the entangled bone morphogenetic protein‐2 (BMP‐2) from DBM^[^ ^41^ ^]^ compared to the particulate DBM controls alone. Overall, the results from this part of this study showed osteoinductivity of the composite and cell‐permeable hydrogel nature of the TX system to keep graft particles in place to support ectopic bone formation. This synergistic effect of the TX carrier system can potentially decrease the required amount of BGMs, while not detrimentally impacting their clinical efficacy.

### 2.2 TX Hydrogel Carrier System for DBM in a Rabbit Femoral Critical Size Defect

A femoral criticalsize cortico‐cancellous defect rabbit model was used to investigate the capability of the TX carrier system to work as intended in a more clinically relevant site. Following the findings from the previous animal study, showing the osteoinductive characteristics of the composite system, TX140/DBM40 was selected for this study as opposed to TX140/DBM70. The selection of the composite with less DBM content (40 vol% in TX140/DBM40 compared with 70 vol% of TX140/DBM70) reduces the false positive effect of DBM and its inherent osteoinductivity. As such, the extent of bone regeneration in sites treated with TX hydrogel and 40 vol% DBM (TX140/DBM40) was compared with 100% DBM (DBM100). To avoid intraspecies inflammatory response in the Oryctolagus cuniculus models, rabbit DBM was used and its osteoinductive properties were confirmed in the standard ectopic animal model prior to the initiation of this part of the study.

**Figure 3:**
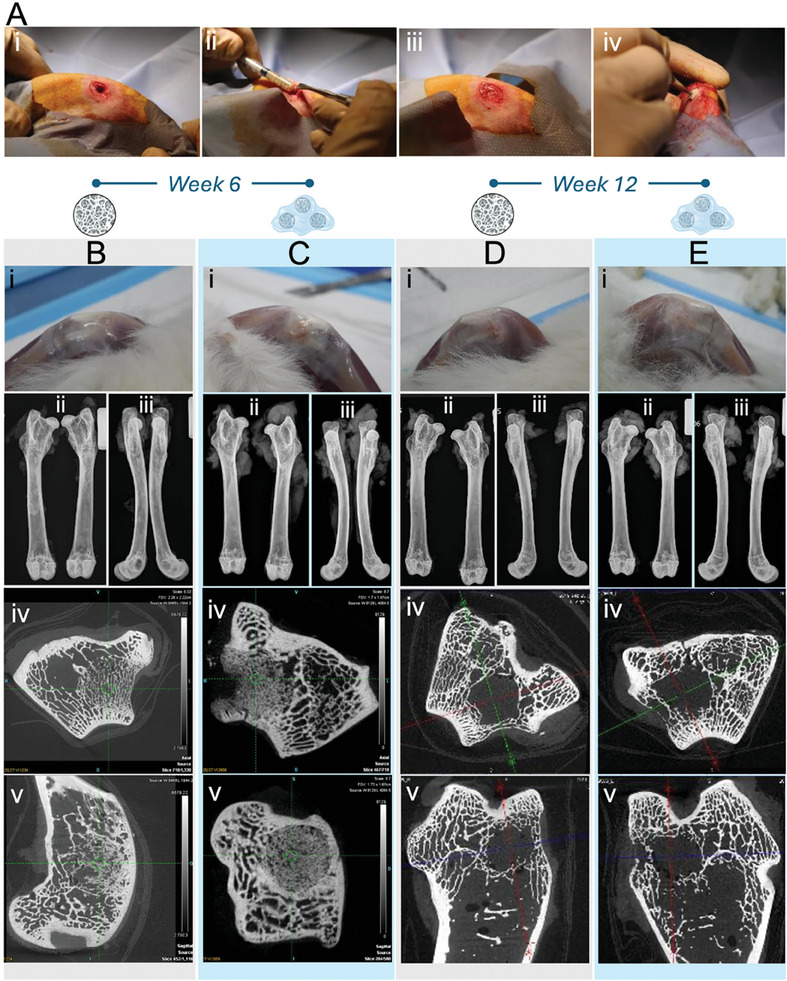
The performance of TX hydrogel carrier for Rabbit DBM implanted within the femur of rabbits. A) Surgical operation on the distal femur of Oryctolagus cuniculus: the defects were drilled with a guide to control the depth i). Following the mixing of the material, the syringe tip was cut to simulate bone graft syringes ii) and the material was directly packed into the defect iii, iv) before closing the tissue in a layered fashion. Treated tissues with B) DBM100 and C) TX140/DBM40 at six weeks post‐implantation. Treated tissues with D) DBM100 and E) TX140/DBM40 at 12 weeks post‐implantation. In panels B–E: i) Subcutaneous tissue at harvest. Faxitron radiographs: ii) anteroposterior and iii) lateral views. MicroCT images: iv) axial and v) sagittal views.

From the handling perspective and as expected, administration of TX140/DBM40 to the site was comparatively easier than DBM100 due to the putty‐like nature of the composite as opposed to DBM particles alone (+Control). The TX140/DBM40 composites could be easily molded to a shape, similar to that of the defect site and subsequently pressed fit to uniformly fill the critical size defect site. In the DBM100 group, however, the particles were adhered to the surrounding soft tissue and needed to be recollected and added to the defect site on multiple occasions, which significantly prolonged the operation time of the sites allocated to the control group. All animals achieved their scheduled termination time points at 6 and 12 weeks post‐surgery. Two groups of animals were sacrificed at these time points. The clinical assessment of the periosteum tissue at the operation sites in panel i) of **Figure** **3**A–E confirmed that at both time points, no sign of infection, inflammation, or any adverse event was observed neither for DBM100 nor TX140/DBM40 treated sites.

The radiographic assessment of the femora at anteroposterior and lateral views of subjects are shown in panels ii) and iii) respectively in Figure 3B–E for both DBM100 and TX140/DBM40. These results for both time points further confirmed the safety of the carrier system. This was concluded as there was no observable difference between the radiographic assessments of the sites treated with the carrier against the control at both time points. To further examine the bone healing progression after 6 weeks, micro‐computed tomography was conducted on the subjects in the axial (Control: Figure 3B‐iv and TX/DBM: Figure 3C‐iv) and sagittal (Control: Figure 3B‐v and TX/DBM: Figure 3C‐v) anatomical planes. Micro‐computed tomography data confirmed the radiographic findings and allowed visualization of the healing defect sites in higher resolution. No adverse reactions to implanted materials were noted. Trabeculae formation is observable at the indicated zones on these images, showing bone healing progression after 6 weeks. The micro‐CT results at 12 weeks post‐administration in the axial (Control: Figure 3D‐iv and TX/DBM: Figure 3E‐iv) and sagittal (Control: Figure 3D‐v and TX/DBM: Figure 3E‐v) anatomical planes, confirmed the results of the radiographic assessment with no apparent sign of infection, favorable cortical bridging and more trabeculae formation at the defect for TX/DBM in comparison with the control group. It should be highlighted that trabecular healing was generally more delayed in the Control group compared to the TX/DBM composite which demonstrated more favorable trabecular healing patterns for the TX/DBM composite. The noticed positive results in TX/DBM might be partially attributed to the better handling of TX/DBM during operation as well as the hydrogel nature of TX, spatially maintaining DBM particles at the defect site. The extent of bone healing at different time points was further investigated via histological assessment of the treatment sites.

**Figure 4:**
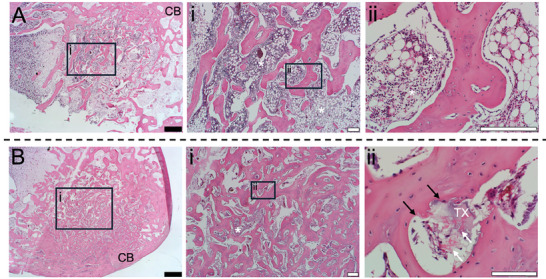
H&E stained of the implanted sites in the femora of the rabbits at 6 weeks post‐surgery. Sites treated with A) DBM100 (+Control) and B) TX140/DBM40. CB: Cortical Bone, TX: Hydrogel remnants. Bone marrow regions are identified with *. White arrows indicate blood vessel ingrowths and black arrows highlight the osteoblasts in the vicinity of TX hydrogel. Scale bars: (Black) 1 mm and (White) 200 µm.

Histological evaluation of the implanted sites with DBM100 at 6 weeks post‐surgery (**Figure** **4**A) demonstrated numerous new bone matrices over the areas of the defect sites and initial signs of bone marrow formation, supported by a meshwork of bone trabeculae inside bones were highlighted by asterisks (*). This result confirmed the radiographic and micro‐CT images, previously outlined. Multiple new bone marrow formation was observed in H&E stained images of the defects at 6 weeks post‐surgery treated by TX140/DBM40 (Figure 4B) similar to the sites treated with rabbit DBM100 (Control). As evident in the histological assessments in Figure 4, bone healing in TX140/DBM40 was not delayed and it progressed at a similar rate to that achieved with DBM100 despite the use of less osteoinductive compounds in the sites treated with the carrier. Interestingly, at the sites treated with TX140/DBM40, the presence of blood vessel ingrowths was apparent (white arrows in Figure 4B‐ii) within the newly generated bony tissue. Moreover, the presence of the osteoblasts in the vicinity of TX hydrogel (Black arrows in Figure 4B‐ii) signifies osteoconductive properties of the hydrogel and its effect on the osteoinductive properties of the composite. These phenomena are due to the tissue‐agnostic regenerative properties of TX140^[^ ^31^ ^]^ which further supports the findings observed in the muscle pouch mice study, indicating that the hydrogel nature of the carrier system positively enhances the effect of DBM for bone regeneration.

### 2.3 Clinical Investigation of TX Carrier in ARP for Trabecular Bone Regeneration

Upon successful completion of the in vivo studies in previous sections and the proven osteoinductivity of TX/BGM and the potential synergistic effect of TX hydrogel with particulate DBM; the clinical utility of TX hydrogels as an osteoconductive bone carrier for trabecular bone regeneration was investigated in a dental extraction socket pilot clinical trial. The study was a two‐armed, randomized, single‐blinded alveolar ridge preservation (ARP) pilot clinical trial involving 11 participants undergoing tooth extraction. TX was used as a carrier system for particulate deproteinized bovine bone mineral (DBBM) Bio‐Oss, Geistlich Pharma AG, Wolhusen, Switzerland, and its handling, safety, and efficacy were compared with particulate DBBM with 10% collagen (DBBM‐C) “control” Bio‐OssCollagen, Geistlich Pharma AG, Wolhusen, Switzerland. Bio‐Oss®Collagen composites contain 90 wt.% DBBM and are hydrated prior to the implantation. To be able to directly compare the handling, safety, and efficacy of TX hydrogel carriers against DBBM‐C, the TX/DBBM composite was formed at the clinic prior to implantation by mixing the TX carrier system with a specific amount of Bio‐Oss granules (0.25 to 1 mm) to achieve a composite with≈90 wt.% DBBM content.

**Figure 5:**
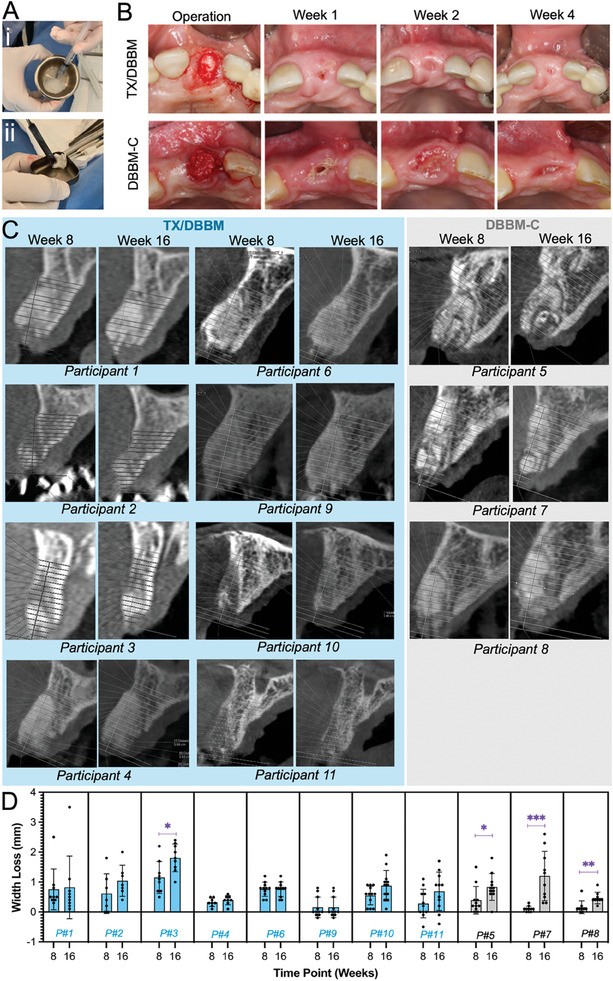
Clinical study of TX/DBBM in ARP post tooth extraction. A) TX/DBBM sample preparation before the operation: i) mixing TX hydrogel with DBBM, and ii) forming a moldable and cohesive putty of TX/DBBM. B) Treatment site at operation (Day 0), and Week 1, 2, and 4 post‐operations of participants with TX/DBBM (TX/ Bio‐Oss®) or DBBM‐C (Bio‐Oss® Collagen) as the Control. Completion of Mucograft ®Seal and tissue epithelization were used to assess the rate and extent of wound closure in participants. C) CT scans of the socket sites treated with TX/DBBM and DBBM‐C at 8 and 16 weeks post‐treatment, the increments (dotted lines) are sliced at 1 mm D) Width loss in the region of interest of the treated sites of all participants treated with TX/DBBM (blue) and DBBM‐C (grey). Data are expressed as mean ± SD. A two‐tailed *t*‐test was performed on means of 8 weeks versus 16 weeks for each patient and the statistical significance was demonstrated as follows: *P < 0.05, **P < 0.01, ***P < 0.001.

Prior to the implantation, TX solution was extruded from its syringe and added to the Bio‐Oss particles, and mixed within 2 min, to form a well‐homogenized TX/DBBM composite (**Figure** **5**Ai). The resulting TX/DBBM composite was moldable allowing a shapable putty to be formed that retained all DBBM particles within the TX hydrogel structure with no separation of the DBBM particles from the composite. TX/DBBM composites were successfully administered to the base of the socket and adhered to the site (Figure 5B) during the entire operative procedure. No device malfunction was noticed throughout the study. In the control group, DBBM‐C was needed to be packed gradually to fill the site layer‐by‐layer. Overall, the TX/DBBM composites could be easily shaped to match the dimension of the extraction sockets using a press‐fitting process which was less time‐consuming compared to the ARP procedure involving DBBM‐C. Following administration of TX/DBBM or DBBM‐C, the extraction sockets were closed by suturing a porcine collagen matrix (Mucograft^® ^Seal, Geistlich Pharma AG, Wolhusen, Switzerland).

All participants treated with TX/DBBM or DBBM‐C returned for the follow‐up visits and their administrated sites were assessed to evaluate the rate of soft tissue healing, presence of Mucograft Seal, and tissue epithelization. The treatment sites at day 0 (operation) and at 1, 2, and 4 week(s) post‐operation are shown in Figure 5B. A summary of findings regarding the safety of the treatment groups is listed in Table S2 (Supporting Information) and the extent of soft tissue healing, and the rate of tissue epithelization are summarized in **Table** **1**. All participants displayed less than 50% tissue epithelization at the site 1‐week post‐operation. After two weeks post‐operation, 6/8 participants treated with TX/DBBM displayed complete tissue epithelization compared to 0/3 in the DBBM‐C control group. Tissue epithelization for all patients was observed 4 weeks post‐operation. Overall, TX/DBBM‐treated patients displayed expedited tissue epithelization compared with the control group (DBBM‐C). Although the sample sizes were small, the relatively faster soft tissue healing profiles in the patients treated with the TX hydrogel carrier may indicate a lower extent of inflammatory response to the implanted material within the underlying TX/DBBM composite.

**Table 1:** Visual assessment of the extent of soft tissue healing and the rate of tissue epithelization.

Participant ID	Age	Material	Week 1 Post‐administration		Week 2 Post‐administration		Week 4 Post‐administration	
			Mucograft Seal Stabilization	Soft Tissue Epithelization	Mucograft Seal Stabilization	Soft Tissue Epithelization	Mucograft Seal Stabilization	Soft Tissue Epithelization
1	71	TX/DBBM	Partial	Partial	Complete	Complete	Complete	Complete
2	83	TX/DBBM	Complete	Partial	Complete	Complete	Complete	Complete
3	29	TX/DBBM	Partial	Partial	Complete	Complete	Complete	Complete
4	47	TX/DBBM	Partial	Partial	Complete	Complete	Complete	Complete
5	22	DBBM‐C	Partial	Partial	Partial	Partial	Complete	Complete
6	60	TX/DBBM	Partial	Partial	Complete	Partial	Complete	Complete
7	26	DBBM‐C	Partial	Partial	Complete	Partial	Complete	Complete
8	52	DBBM‐C	Partial	Partial	Partial	Partial	Complete	Complete
9	80	TX/DBBM	Partial	Partial	Complete	Complete	Complete	Complete
10	37	TX/DBBM	Partial	Partial	Complete	Complete	Complete	Complete
11	47	TX/DBBM	Partial	Partial	Complete	Partial	Complete	Complete
**Summary**		TX/DBBM Group	1/8	0/8	8/8	6/8	8/8	8/8
		DBBM‐C	0/3	0/3	1/3	0/3	3/3	3/3

CBCT scans of the maxilla at 8 and 16 weeks post‐operation are shown in Figure 5C. The sites are sectioned, and the regions of interest are identified using a standardized method as described in Supporting Information (Figures S6 and S7, Supporting Information). In all participants, the socket widths at each slice (dotted lines) were compared with corresponding pre‐operation measurements to calculate the loss in bone width at 8 weeks and 16 weeks post‐operation. Subsequently, the measurements of the width loss in the region of interest were performed (Figure 5D). Overall, there was no significant difference in average bone loss at the region of interest between the TX/DBBM test group (0.82 ± 0.50 mm) and the DBBM‐C control group (0.83 ± 0.37 mm) after 16 weeks. The average horizontal bone loss at the site treated with TX/DBBM and the control group was less than 1 mm in width. Bone loss in the Control group, treated with DBBM‐C was expected and is similar to other published reports of this product which is widely used for ARP.^[^ ^42^, ^43^, ^44^ ^]^ The similar width loss in the patients treated with the TX/DBBM showed the clinical utility of TX and its potential as a carrier of DBBM particles and potentially other classes of particulate BGM that have also been used in ARP procedures.

More importantly, CT results depicted in Figure 5C showed that the quality of trabecular bone at the sites treated with TX/DBBM was deemed more uniform compared with DBBM‐C administered sites. It's worth noting the difference in CBCT trabecular homogenization patterns between the TX/DBBM groups and the DBBM‐C group, indicating a more uniform radiopaque trabecular bone pattern for those sites treated with TX/DBBM compared to the DBBM‐C control (Figure 5C). This finding suggests that DBBM particles are more evenly homogenized within the TX carrier system (TX/DBBM) compared with the control (DBBM‐C). This result was aligned with the clinical observations noticed during the administration of the two composites, confirming the enhanced handling characteristics of the composites with TX hydrogel and its potential to allow gentle press fitting of the composite for complete filling of the site. In addition, results in Figure 5D showed that 1/8 participants in the TX/ DBBM group and 3/3 participants in the control group displayed significant (*P* < 0.05) bone loss between 8 and 16 weeks. Hence, it is inferred that in the TX/DBBM treated group, bone resorption achieved its peak and plateaued at earlier stages post‐surgery compared with DBBM‐C. This result may suggest a more uniform pattern of tissue integration is occurring within the TX carrier system compared to the collagen matrix used in the control. As such, the effect of the TX hydrogel on the extent of trabecular bone ingrowth and foreign body reaction at the sites was histologically evaluated.

After 16 weeks post administration of TX/DBBM or DBBM‐C, all participants underwent implant placement. Although all implants were fully contained within the regenerated trabecular bone, additional horizontal bone augmentation to provide improved contour aesthetics was undertaken on one control patient and 3 experimental patients (Figure S8, Supporting Information). The rationale for this additional horizontal bone augmentation procedure at the time of implant placement has been previously described in the literature for ARP involving DBBM‐C.^[^ ^42^ ^]^ Results indicated that socket preservation was successful in all participants regardless of their treatment allocation which supports the clinical potential of TX as a hydrogel carrier system for in situ mixing and delivery of different bone grafts. Immediately prior to the implant placement, bone specimens were collected using a 2 mm trephine drill for histochemical analyses.

**Figure 6:**
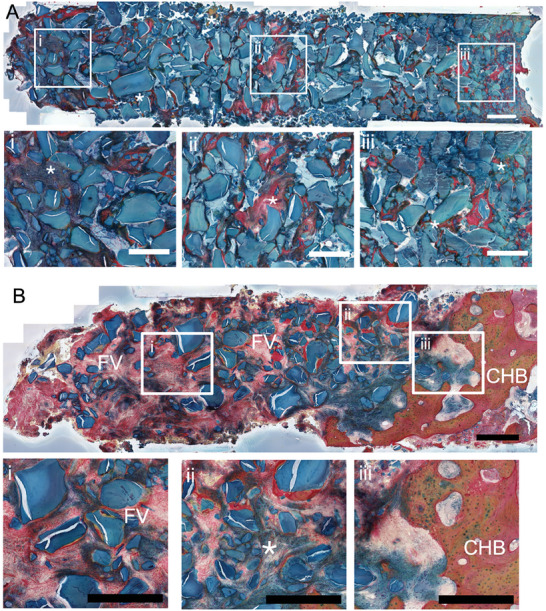
Movat Pentacrhome stained trephine specimens from the implantation sites of the participants. Sites treated with (A) TX/DBBM and (B) DBBM‐C. FV: fibrovascular tissue, CHB: Cortical host bone. Trabecular bone integration within DBBM particles is indicated with *. Scale bars = 500 µm.

Movat pentachrome staining of the sites was used to assess the biological interaction with TX/DBBM and DBBM‐C, (**Figure** **6**). In movat pentachrome stained section DBBM particles are acellular structures that are stained blue, trabecular bone is light green/yellow, cortical host bone is light orange, and fibrovascular tissue is red. In the control group, treated with Bio‐Oss Collagen, the DBBM particles were scattered, and their non‐uniform distribution within the site was noticed (Figure 6A). The granules were entangled with some degree of trabecular bone and fibrovascular tissue (stained red). The non‐uniform structure of the treatment site and their entanglement within the trabecular bone and fibrovascular tissue were aligned with findings from soft tissue healing observations and CT scan results in the Control group, showing a heterogeneous trabecular bone pattern. Conversely, in TX/DBBM treated sites, the DBBM particles were uniformly distributed within the socket (Figure 6B), confirming the potential of the TX hydrogel and its structural stability to retain graft particles spatially in a 3D manner, which is essential for the integration of the graft with the host environment. As such, trabecular bony tissue ingrowth, stained light yellow/green was noticed throughout the area treated with TX/DBBM. In addition, the extent of the inflammatory response to the TX hydrogel was minimal at the implantation site, evident by the absence of fibrovascular tissue at the site. All patients returned back to their referring dental practitioners for implant restoration restored 3–5 months following implant placement. All implants were successfully restored at this time point with 1‐year post‐restoration assessment demonstrating an implant survival rate of 100%.

## 3 Discussion

The delivery of particulate BGM in the form of putty can result in a less cumbersome surgical application to form a homogenous structure and potentially avoid surgical complications attributed to graft migration. In these series of in vivo animal and human clinical studies, we have been able to demonstrate that the TX hydrogel, can be used with two classes of particulate BGMs for bone defects involving both the trabecular and cortical spaces. The hydrogel nature of TX system keeps graft particles in place and retains the osteoinductive properties of the composite to support ectopic bone formation, as verified in the nude murine study. The synergistic effect of the TX carrier system can potentially decrease the required amount of particulate DBM, while not detrimentally impacting the osteoinductivity of the composite. This observation was further supported in the femoral critical‐size cortico‐cancellous defect rabbit model, where the TX/DBM composite with less DBM content displayed similar bone regenerative properties to that observed in the control with 100% DBM content.

When TX was used as a carrier for DBBM in the ARP clinical procedure involving trabecular bone regeneration, bone resorption achieved its peak and plateaued at earlier stages post‐surgery. In this respect, it was observed that 12.5% (n = 8) of the investigational group treated with the fully synthetic TX carrier for DBBM demonstrated further horizontal alveolar ridge resorption between 8 and 16 weeks, compared with 100% (n = 3) of the control group treated with DBBM‐C which underwent further horizontal alveolar ridge resorption. It should be acknowledged that this was a limited pilot study to primarily assess the handling, safety, and cellular inflammatory activity of TX with particulate DBBM in human extraction sockets. In this respect, the sample size is such that powered statistical analysis was not possible. The results of this pilot study, however, will be used to inform the design of future expanded single‐center and multi‐center clinical trials.

TX hydrogel, as the carrier for BGMs, was compared to other BGMs carriers, *e.g*., collagen, glycerol, carboxymethyl cellulose, poloxamer, and hyaluronic acid (in the form of sodium hyaluronate) that have been used commercially to deliver BGMs in putty form (**Table** **2**). One main feature of TX carrier is its modularity which in this study has been used successfully at various concentration ratios with different types of BGMs (human‐ and animal‐derived BGMs). This is in contrast to other carrier vehicles that can only be used with specific bone graft particles with a fixed amount of BGMs. This modularity has the potential to expand the applications of TX to be used in various bone defects unlike some of the commercial products, listed in Table 2, that are limited to a certain type of bone defects.

**Table 2:** Comparison of carriers in some commercial products (putty form) against the material used in this work.

Carrier	Commercial Example	BGM	Application	Degradation Rate of The Carrier	Immune Response	Challenges
Collagen	OsteoBiol® Putty (Tecnoss, Italy)	Heterologous cortico‐cancellous bone mix	Used in periodontal bone regeneration, orthopedic bone repair.	‐ Collagen degrades in a few days.^[^ ^45^ ^]^ ‐ The degradation rate of the whole device (BGM included) depends on the source of the BGM and took from 6 months to 12 months post‐implantation.	‐ Inflammatory response due to degradation of the collagen membrane.^[^ ^45^ ^]^ ‐ Slight immune response: collagen promotes osteoconduction and angiogenesis.^[^ ^45^ ^]^	‐ Potential variability in porcine collagen purification affecting osteoconductivity and biocompatibility.^[^ ^45^ ^]^ ‐ Collagen sources can raise immunogenic concerns.^[^ ^45^ ^]^
	Bio‐Oss® Collagen Putty (Geistlich Pharma, Switzerland)	90% Geistlich Bio‐Oss micro granules (bovine)	Primarily used for dental bone grafting, periodontal defects, sinus lift procedures.			
	MinerOss® Putty (BioHorizons, USA)	50:50 ratio of cortical and cancellous bone chips	Indicated for periodontal bone regeneration, dental implant procedures, and orthopedic use for bone defects.			
Glycerol	Grafton™ DBM Putty (Medtronic, USA)	DBM	Orthopedic or reconstructive bone grafting procedures. bone grafting procedures in combination with autologous bone.	‐ Glycerol is a hydrophilic, small molecule that is unstable with fast resorption in body^[^ ^46^ ^]^ (≈a few days).	‐ Glycerol in Grafton is toxic in large‐scale use and the dose should not exceed 2 mL kg^−1^, clinically.^[^ ^49^ ^]^	‐ Should be used carefully in treatment of smokers with bone graft.^[^ ^51^ ^]^ ‐ There are still some controversies about its use due to its unstable and potentially toxic in large‐scale of glycerol.^[^ ^46^ ^]^
Poloxamer		Accell Evo3® DBM Putty (Integra, Plainsboro, USA)	DBM ≈40v%	Intended to use as a bone graft extender in the spine, extremities and pelvis or as a bone void filler for the extremities and pelvis.	‐ Low stability of these carrier systems^[^ ^52^ ^]^ does not provide a matrix to preserve the DBM granules in a 3D manner.	‐ The poloxamer itself has no toxicity,^[^ ^53^ ^]^ but it may inhibit the osteoblastic differentiation by filling up the spaces between the DBM granulates, which negatively affects the release of growth factors.^[^ ^50^ ^]^	‐ Reduced growth factor release.^[^ ^50^ ^]^
Carboxymethyl Cellulose (CMC)	C‐Blast Putty™ (Citagenix Inc, Canada)	Osteoinductive DBM with osteoconductive cancellous bone particles	Periodontal defects, Coronal defects around immediate implants, Extraction site repair, Implant dehiscence defects, Sinus lift, Moderate localized ridge defects.	‐ Degrades within a few days or weeks depending on the environment.^[^ ^47^ ^]^	‐ CMC in Calstrux used in combination with other products has resulted in a trend in adverse event reports including localized induration, swelling, inflammation, wound drainage, infection and device migration (*Recall Event ID: 36 089*).	‐ Rapid degradation can outpace host bone formation. ‐ Carboxymethyl Cellulose does not display 3D structural properties, and no supportive impact on the bone regeneration.^[^ ^48^ ^]^
	Calstrux (TCP Putty, Stryker Bioteh, USA)	Tri‐calcium phosphate granules	Intended as bone filler to be packed in bony voids in extremities, spine, and pelvis).			
Hyaluronic acid (sodium hyaluronate)	DBX® Inject Putty (Johnson & Jonson, USA)	DBM (Cortical bone)	It is indicated for treatment of surgically created osseous defects or osseous defects created from traumatic injury.	‐ Hyaluronic acid degrades fast, at five days.^[^ ^54^ ^]^	‐ Minimum inflammation.^[^ ^55^ ^]^	‐ Fast degradation of the carrier hinder the usability of the products against other competitors. ‐ The rate at which the carrier biodegrades and integrates into native bone tissue varies.
TX hydrogel *(This Work)*	N.A.	DBM or DBBM with various sources	Intended to be used for all bone defects.	‐ TX hydrogel is stable without any change in mass for at least 14 days.	Minimum inflammation as the result of tissue regeneration.	‐ More clinical trials with larger sample size and for other bone defects are needed.

Another limitation of most particulate BGM carriers used in commercial devices is their high rate of degradation or their low stability; for instance, collagen, glycerol, poloxamer, and hyaluronic acid either resorb or degrade within a few days.^[^ ^45^, ^46^ ^]^ Such fast degradation of these carriers leads to structural collapse of the implanted products and inhomogeneous distribution of the graft particulates within the intended anatomical site. Carboxymethyl cellulose‐based products are more stable for a longer period^[^ ^47^ ^]^ however, carboxymethyl cellulose does not display 3D structural properties which limits its ability to spatially support the particulates for bone tissue integration.^[^ ^48^ ^]^

With regard to safety and efficacy, there are some concerns about the inflammation and toxicity of some of the commercial carriers listed in Table 2. The synthetic nature of TX hydrogel mitigates the risks associated with natural carrier systems such as collagen which are mainly attributed to their intraspecies sources and impurities.^[^ ^45^ ^]^ Glycerol may cause toxic reactions in large‐scale.^[^ ^46^, ^49^ ^]^ While other commercial carriers are deemed to be non‐toxic themselves, they may cause inflammatory responses or detrimentally impact the activity of incorporated BGMs. For example, poloxamer has been shown to decrease the activity of DBM granules.^[^ ^50^ ^]^ Another example is carboxymethyl cellulose which in combination with other products has resulted in adverse events including localized induration, swelling, inflammation, wound drainage, infection, and device migration which resulted in the recall of the product by the FDA (Recall Event ID: 36089). In comparison to other commercially available carrier vehicles for particulate BGMs that have been used in putty form, TX has higher physical and mechanical stability.

## 4 Conclusion

This study has demonstrated the efficacy of TX hydrogel as a universal bone graft carrier through in vivo preclinical and clinical studies. It is inferred that the TX hydrogel carrier supports osteoinduction, is osteoconductive, and has minimal foreign body reactions at the treated sites post‐administration. This work successfully illustrates the utility of the TX hydrogel as a carrier system for human‐ or animal‐derived demineralized bone matrix as well as deproteinized bovine bone mineral. Upon mixing with the carrier system, the particulate bone graft materials were fully adherent to and contained within the TX hydrogel. The resulting composite putty was easy to handle, permitting a simplified clinical application. In summary, the unique physical characteristics of the TX hydrogel carrier system allow particulate bone graft materials to be spatially separated in a 3D manner resulting in improved handling, minimal hard and soft tissue inflammatory responses and favourable cortical and trabecular bone regenerative outcomes.

## 5 Experimental Section

### Hydrogel‐Based Carrier Synthesis

Poly(*N*‐isopropylacrylamide‐*co*‐polylactide‐2‐hydroxyethyl methacrylate‐*co*‐oligo (ethylene glycol) monomethyl ether methacrylate‐*co*‐*N*‐acryloxysuccinimide), denoted as PNPHO, was synthesized through radical polymerization at Tetratherix (Sydney, Australia) as described, previously.^[^ ^31^, ^33^ ^]^ Briefly, the molar ratio of the constructing monomers including *N*‐isopropylacrylamide, *N*‐acryloxysuccinimide, polylactide/‐hydroxy methacrylate (lactate number of 5), and oligo (ethylene glycol) were 81:7:7:5 in dimethyl formamide with a solid content of 16 w/v%. The reaction was initiated by adding 4,4′‐Azobis (4‐cyanopentanoic acid) to the solution at 70 °C. After 18 h, the crude polymer solution was collected, purified, and lyophilized. The resulting white PNPHO powder was used to form TX140 and TX280 by conjugating the polymer with a synthetic peptide, Thymosin ß4 (Tß4) as outlined previously.^[^ ^31^ ^]^ The subsequent single‐phase PNPHO‐Thymosin‐ß4 solutions were sterilized using gamma irradiation at 25 – 40 kGy at Steritech NSW (Australia) to achieve a SAL of 10^−6^ per ISO 11137‐1, ISO 11137‐2, ISO 11137‐3 and EN 556‐1.

### Nude Mice Hind Limb Osteoinductive Study

Human‐derived DBM (Boost Bone Matrix™) powder was obtained from Australian Biotechnology Pty Ltd. In this standard osteoinduction muscle pouch study (n = 24, Balb/c nude mice 10–12 weeks of age and with an average weight of 22.75 ± 1.13 g and two implantation sites: i) right limb and ii) left limb per animal), ≈100 µl of test materials were implanted at each hind limb site. Four different configurations (Groups), including i) TX280/DBM20, ii)TX140/DBM40, iii) TX140/DMB70, and iv) DBM100 were prepared, randomized, and implanted in the mice (two incisions per animal). These mice were separated into 2 timepoint groups: 12 mice in week 4 and 12 mice in week 6 groups. The digits after DBM refer to the volume ratio of DBM:TX. For example, TX140/DBM70 refers to a composite of TX140 with 70 vol% DBM and 30 vol% TX140. All test materials, prepared with TX140 or TX280 were flowable and therefore directly implanted within the muscle pouch site. The +Control group, DBM100 was pre‐wetted and packed into the muscle pouch via a spatula. Each experimental mouse was housed in a single standard cage with free access to food and water at the Translational Research Facility of the ANZAC Research Institute, Sydney, where the environment was controlled at 24–26 °C and 44%–46% humidity under a 12:12 h light:dark cycle. Mice were given post‐operative analgesics, carprofen (5 mg kg^−1^), on the day of the procedure and in 24 h intervals for 2 days post‐procedure under the ethics protocol.

For week 4 group, implanted hind limbs of mice were scanned with Dual‐Energy X‐ray Absorptiometry (DEXA; PIXImus2 Series Densitometer, GE Healthcare, USA) weekly and X‐rays in week 2 and week 4 post‐procedure using a Faxitron (150 VAMAX, Model #MX‐20, Auburn, CA, USA; settings 50 s and 20 kv). All mice in this group were anesthetized with ketamine:xylazine (100 mg:100 mg kg^−1^) and euthanized by cervical disclosure 4 weeks after the procedure. In the week 6 group, the hindlimbs of mice were scanned using DEXA every week and x‐rayed in weeks 3 and week 6 post‐procedure. All mice in this group were euthanized 6 weeks post‐procedure. Following euthanasia, mouse hindlimbs were harvested for micro‐CT analysis and histological analyses. The volume of the newly formed bone tissues was analyzed by software, CTAn (CT‐Analyzer, Bruker BioSciences Pty Ltd, USA). Following CT analysis, the bone samples were demineralized and processed for histological analysis.

For the histological assessments, the hindlimbs involving the femur area with surrounding muscle tissues were fixed in 10% formalin for 24 h and transferred to the PBS solution before imaging with a high‐resolution micro‐CT system (Skyscan 1172 instrument, Bruker MicroCT, Kontich, Belgium). The harvested mouse hindlimbs were decalcified in 10% Ethylenediaminetetraacetic acid, EDTA (changed 3 times a week) solution, pH 7.5 for 3 weeks at 4 °C. Tissues were then processed in a wash cycle of ethanol, xylene, and paraffin, embedded in paraffin, and sectioned at a thickness of 5 µm. Sections were stained with hematoxylin and eosin (H&E) for analysis of ectopic bone histology. This animal study was approved by the Institutional Animal Ethics Committee, ANZAC Research Institute (protocol number: 2017/026C).

### Rabbit Femoral Critical Size Defect Model

Nine female New Zealand white rabbits (Oryctolagus cuniculus) were selected each with a body weight of greater than 3.5 kg, and each was greater than 7 months of age with skeletally mature as verified by growth plate closure. The age of the animals had been chosen so that animals were likely to be at or close to skeletal maturity. Skeletal maturity was assessed and confirmed prior to inclusion in the study. The nine rabbits were distributed in two time point groups: i) 5 rabbits in 6 week group and ii) 4 rabbits in 12 weeks group. Prior to the initiation of the study, rabbit‐derived DBM was developed by using rabbit long bones. The osteoinductivity of the processed rabbit DBM was verified by using the standard hind limb osteoinductive mice model, previously described. 18 surgically created, critical size defects were formed in the right and left femora of nine female New Zealand white rabbits (Oryctolagus cuniculus). Bilateral defects (6 mm in diameter and 10 mm deep) were created in cancellous bones, using a 6 mm drill and depth indicator. Defects of this size, 4–6 mm diameter and 10 mm deep in the distal femur in animals of the same age and younger have been reported to be critical.^[^ ^56^, ^57^ ^]^ Animals were implanted with TX140/DBM40 and DBM100 as the control. The skin was opened, and the periosteum was reflected using a periosteal elevator in the medial aspect of the distal femur. The defects were prepared under saline irrigation to minimize thermal damage. The defects were flushed with sterile saline during the preparation and at the completion to remove any residual bone. TX140/DBM40 were directly administered from syringe bores to the site whereas the control group was placed in the defects using a spatula to the height of the original cortex. This animal study was approved by the Animal Care and Ethics Committee at the University of New South Wales (19/86A, TRP2019‐1).

The right and left femora were harvested and photographed using a digital camera at their designated termination time points (6 or 12 weeks post‐implantation). The harvested femora were radiographed using Faxitron (Faxitron Bioptics, USA) and high‐resolution mammography film (settings 24 kV for 45 s) and recorded on digital plate sensors (AGFA CR 75.0 Digitizer Musica, AGFA, Germany). The DICOM data was converted to Bit‐map images using DICOM Works (ezDICOM medical viewer, copyright 2002). The Faxitron radiographs were evaluated for defect positions as well as for any gross adverse bony reactions. Micro Computed Tomography (CT) was performed on all femora using an Inveon in vivo microcomputer tomography scanner (Siemens Medical, PA, USA). The femora were scanned, and the images were examined in the axial, sagittal, and coronal planes to assess the healing at the implantation sites and examined for any adverse events. The slice thickness was 44 and 500 µm, taken at the edges of the defect and in the middle of the defect.

The harvested distal femora were fixed in 10% phosphate‐buffered formalin at room temperature with gentle rotation on a Labtech rotating shaker, for a minimum of 96 h. Paraffin histology was performed following the standard operating procedure for distal femur defects. The samples were decalcified in 10% formic acid – phosphate‐buffered formalin at room temperature. Samples were decalcified in this solution 3–4 days before gross sectioning into paraffin cassettes. The cut sections (≈3 mm in thickness) were placed into embedding blocks for paraffin processing from medial to lateral in the sagittal plane. Each paraffin block was sectioned (5 microns) using a Leica Microtome and placed on slides for Haemotoxylin and Eosin (H&E) staining (see Supporting Information document).

### Tooth Extraction Socket Alveolar Ridge Preservation Clinical Study

Ethics approval for this clinical study was granted by St. Vincent's Hospital, Melbourne, Australia Ethics Committee (ref HREC 043/19, project ID: 52231) prior to patient enrolment and performed in accordance with the principles established in the Declaration of Helsinki. The trial protocol was registered prospectively with the Australia and New Zealand Clinical Trials Registry (ANZCTR): 12619001124123. Written and verbal informed consent was provided to all subjects. The details of the clinical study were provided in the Supporting Information document.

A standard alveolar ridge preservation (ARP) clinical trial was designed (see the supplementary information for more details) in which the investigational product (TX) was combined with particulate DBBM (small particle, cancellous, 0.25–1 mm, Bio‐Oss, Geistlich Pharma AG) to assess the clinical usability, safety, and efficacy of the TX hydrogel carrier. To this end, the TX carrier system was mixed intraoperatively with Bio‐Oss, a DBBM which is widely used for alveolar trabecular regenerative procedures. For the comparator control (+ control), DBBM combined with a 10% collagen matrix (DBBM‐C) (Geistlich Bio‐Oss Collagen; Geistlich Pharma AG) was used. Bio‐Oss Collagen was a ready‐to‐use DBBM and porcine collagen composite that was widely used by clinicians for ARP and has been extensively reported in the literature. Patients were randomly allocated to the investigational device (DBBM/TX) or the +Control (DBBM‐C).

Alveolar ridge preservation was then performed. For the TX/DBBM group, the TX carrier was mixed chairside with DBBM for 2 min prior to implantation. The resulting composite was manipulated, so the final composite involved ≈90 wt.% DBBM and 10 wt.% TX. The resulting TX/DBBM composite was then gently packed into the base of the socket post‐tooth extraction to the level of the alveolar bone crest. For the control group, the DBBM‐C porous block was hydrated with isotonic sterile saline, cut into fragments, and packed into the base of the post‐tooth extraction to the level of the alveolar bone crest. For all participants, the socket entrance was closed with a resorbable type I/III porcine collagen matrix (CM) (Geistlich Mucograft Seal; Geistlich Pharma AG) that was sutured into position using interrupted 5‐0 polyglactin sutures (Novosyn Quick, B/Braun, Surgical, SA. Rubi Spain). Post‐surgical oral antibiotics were prescribed for all participants, regardless of their designated treatment group: Amoxicillin 500 mg tds (6 days) or Cephelaxin 500 mg tds (6 days) or Clindamycin 150 qid (6 days) if there was a known anaphylactic reaction to penicillin. Paracetamol 500 mg tds or Paracetamol 500 mg combined with Codeine Phosphate 30 mg was provided for inflammation and pain (if required).

Analytically, 8 weeks and 16 weeks post‐operation, cone beam computerized tomography (CBCT) imaging was performed to assess the post‐extraction bone volume and trabecular healing patterns at the former extraction sites. In the final follow‐up visit (16 weeks post extraction/BGM administration), patients underwent dental implant surgery during which post‐extraction regenerated trabecular bone was sampled with a 2 mm trephine drill for histochemical assessment. The collected specimen was placed in a pre‐filled pathology fixation kit with formalin. The fixed specimen was sent to an independent pathology laboratory for hematoxylin and eosin (H&E) and Movat's pentachrome staining and analyses. Subsequently, bone remodeling, osteoblast and osteoclast activities, and TX interactions with DBBM were compared with the control group at 16 weeks post‐surgical timepoint. Cone beam computerized tomography (CBCT) measurements were performed using a standardized protocol at 3 time points: pre‐extraction, 8 weeks post‐extraction, and 16 weeks post‐extraction. Other details regarding this clinical trial including inclusion and exclusion criteria, patient demographics, clinical procedures, and socket preservation measurements can be found in the Supporting Information.

### Data Analysis

Descriptive analysis was applied to all data and the mean ± standard deviation was expressed as the quantitative values in all charts. One‐way ANOVA followed by the Tukey test (as post‐HoC analysis) was conducted where there were more than two groups. For comparing the means of two groups (investigational device group versus control group) a two‐tailed *t*‐test was applied. Statistical significance was set at *P* < 0.05 for all statistical analyses. Statistical significance was demonstrated on all charts as * for *P* < 0.05, ** for *P* < 0.01, and *** for *P* <0.001. Prior to means comparison testing, the Shapiro‐Wilk test was performed to assess the normality of the data. Before performing a two‐tailed *t*‐test, an *F*‐test was performed to test the equality of each two variances. All statistical analyses were conducted using GraphPad Prism version 10.

## Conflict of Interest

S.M., T.A., A.O., F.O. and A.F. are employed by Tetratherix. A.F. and F.D. are co‐inventors in AU2016301103; EP16829490.8; JP2018‐503480; US15/746810; U2016314146; EP344304; JP2018‐529692; US 17/090078; EP 2794701; US9546235. A.F. and T.A. are co‐inventors in PCT/AU2020/051332 and the Chinese national phase patent application (2020800006185.0). D.C. is a clinical advisor to Tetratherix. All other authors declare no competing interest.

## Author Contributions

D.C. and F.O. contributed equally to this work. D.C. performed co‐conceptualization methodology (surgical procedures in the clinical trial), validation (clinical trial), and wrote the original draft. F.O. performed wrote the original draft and visualization. S.M. performed wrote, reviewed, edited, and methodology. T.A. performed project administration, wrote, reviewed and edited. T.H. and B.K. developed the CBCT method for ridge width quantification in the clinical study and performed formal analyses (analytical imaging) and wrote, reviewed and edited. A.O., W.C._,_ and F.D. performed wrote, reviewed and edited. A.F. performed co‐conceptualization, methodology, wrote, reviewed and edited, funding acquisition and supervision.

## Supporting information



Supporting Information

## Data Availability

The data that support the findings of this study are available on request from the corresponding author. The data are not publicly available due to privacy or ethical restrictions.
