# Supplementary material for: Universal Hydrogel Carrier Enhances Bone Graft Success: Preclinical and Clinical Evaluation
Source: Adv Healthc Mater. 2025 Jan 22;14(12):2403930. doi: 10.1002/adhm.202403930 (PMC12057595; doi:10.1002/adhm.202403930)
Supplement: Supplementary file 1 — Supporting Information [file ADHM-14-0-s001.docx]

Supporting Information

Universal Hydrogel Carrier Enhances Bone Graft Success: Preclinical and Clinical Evaluation

*D. Calder^a,b,1^, F. Oveissi^c,d,1^, S. Maleknia^d^, T. Huang^e^, B. Koong^e^, T. Abrams^d^, A. Oar^d,f^, W. Chrzanowski^g,h,a^, F. Dehghani^c,^* and A. Fathi^c,d,^**

^a^ Sydney Pharmacy School, Faculty of Medicine and Health, University of Sydney, Sydney, NSW, 2006, Australia

^b^ Dental School, University of Western Australia, Perth, WA, 6009, Australia

^c^ School of Chemical and Biomolecular Engineering, The University of Sydney, Sydney, NSW 2006, Australia

^d^ Tetratherix Technology Pty Ltd, Sydney, NSW, 2000, Australia

^e^ Envision Medical Imaging, Wembley, WA, 6014, Australia

^f^ Gold Coast University Hospital, Southport, 4215, Australia

^g^ Department of Laboratory Medicine, Division of Biomolecular and Cellular Medicine, Division of Clinical Immunology, Karolinska Institute

^h^ Division of Biomedical Engineering, Department of Materials Science and Engineering, Uppsala University

^1^ These authors (D.C. and F.O.) contributed equally to this work; co-first authors.

*Corresponding author:

A.F. (E-mail: [ali.fathi@sydney.edu.au](mailto:ali.fathi@sydney.edu.au); [ali.fathi@tetratherix.com](mailto:ali.fathi@tetratherix.com))

F.D. (E-mail: [fariba.dehghani@sydney.edu.au](mailto:fariba.dehghani@sydney.edu.au))

# H&E Stained of implanted sites in nude mice study 4 weeks post-operation


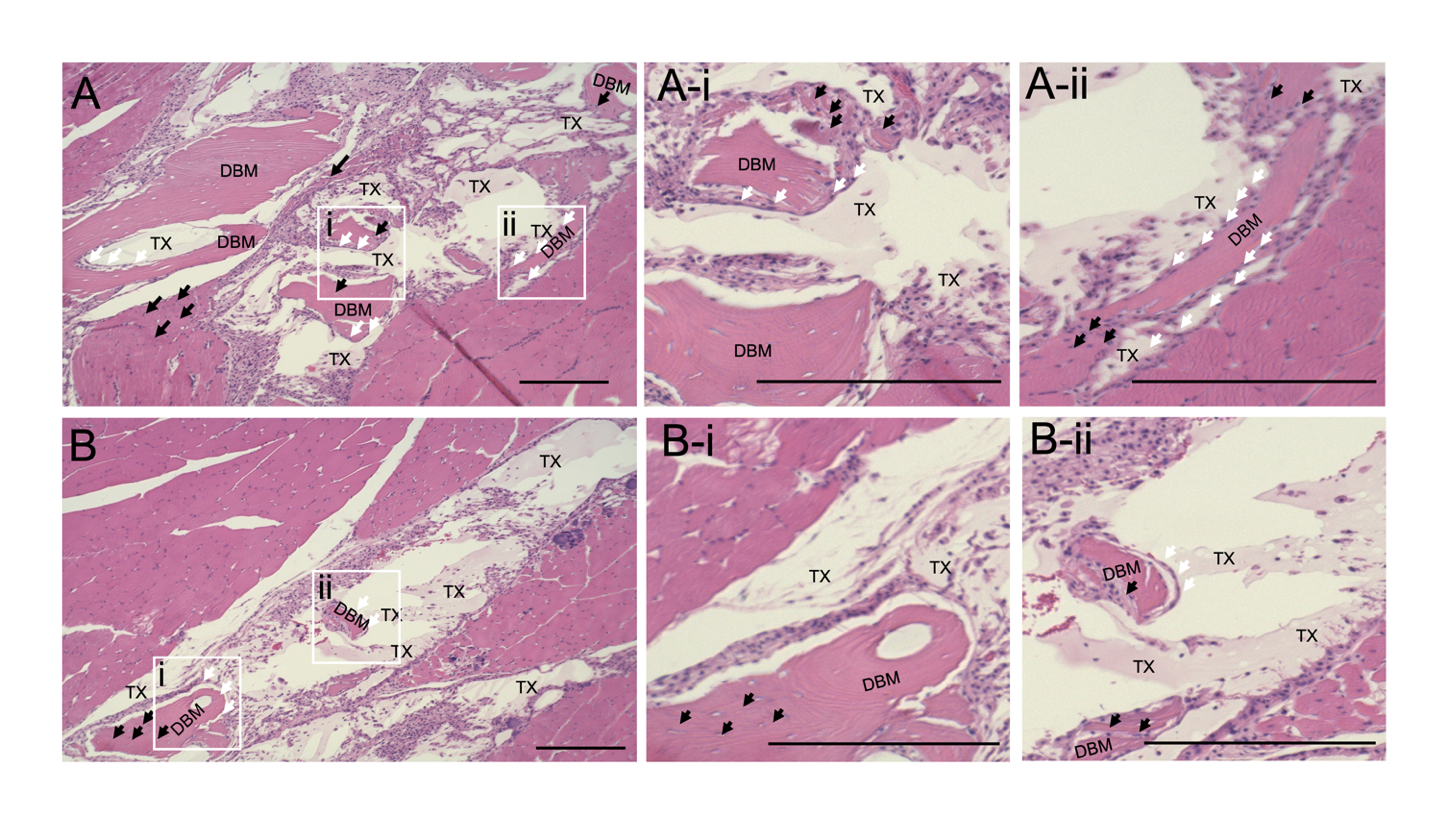


**Figure S1.** **H&E Stained of implanted sites with TX280/DBM20, 4 weeks post-implantation in hindlimbs of nude mouse models.** TX: hydrogel remnants, white arrows show osteoblast cells in the vicinity of hydrogel and DBM granules, black arrows show osteocytes. Scale bars are at 100 µm.


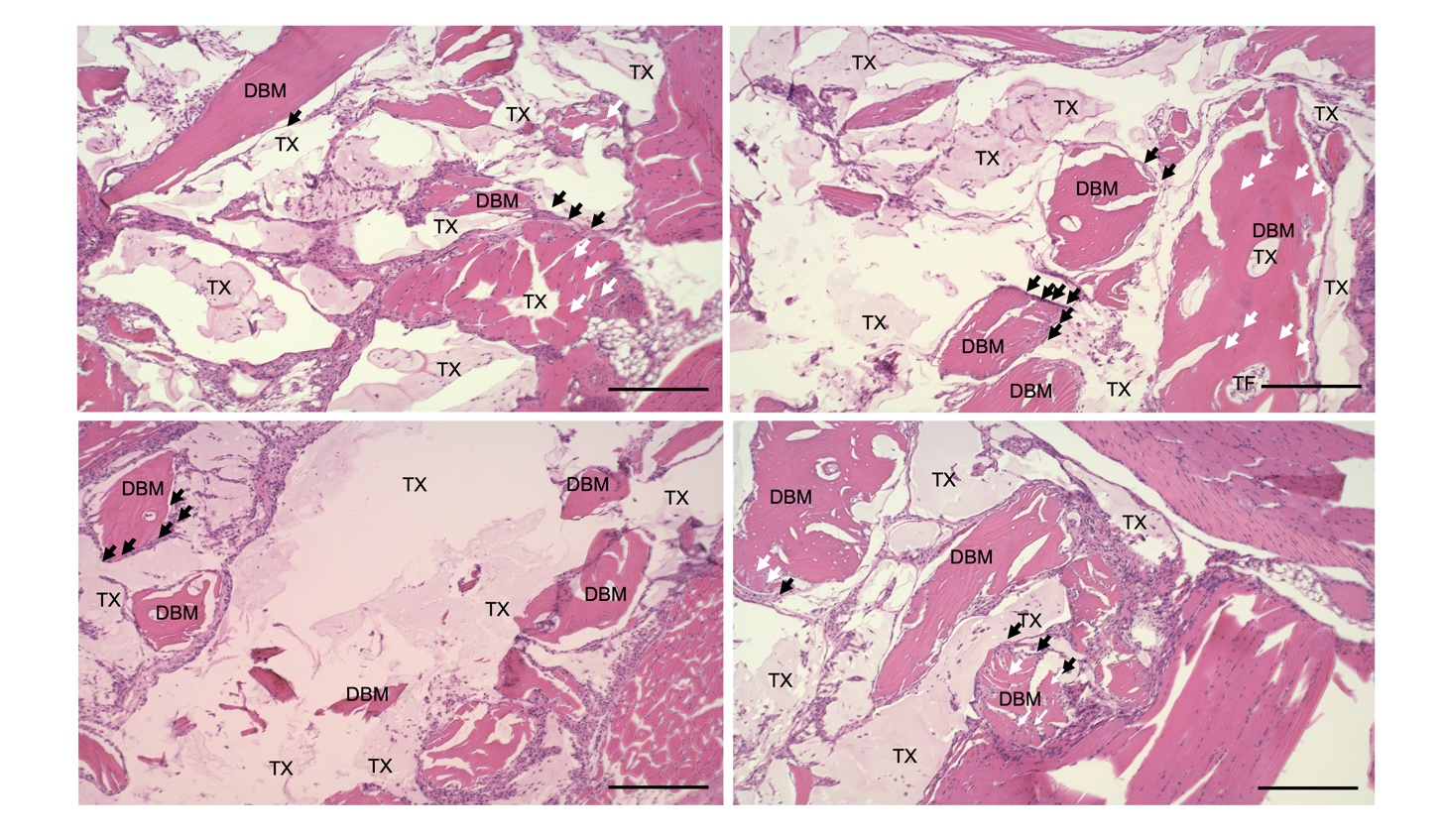


**Figure S2.** **H&E Stained of implanted sites with TX140/DBM40, 4 weeks post-implantation in hindlimbs of nude mouse models.** TX: hydrogel remnants, white arrows show osteoblast cells in the vicinity of hydrogel and DBM granules, black arrows show osteocytes. Scale bars are at 100 µm.


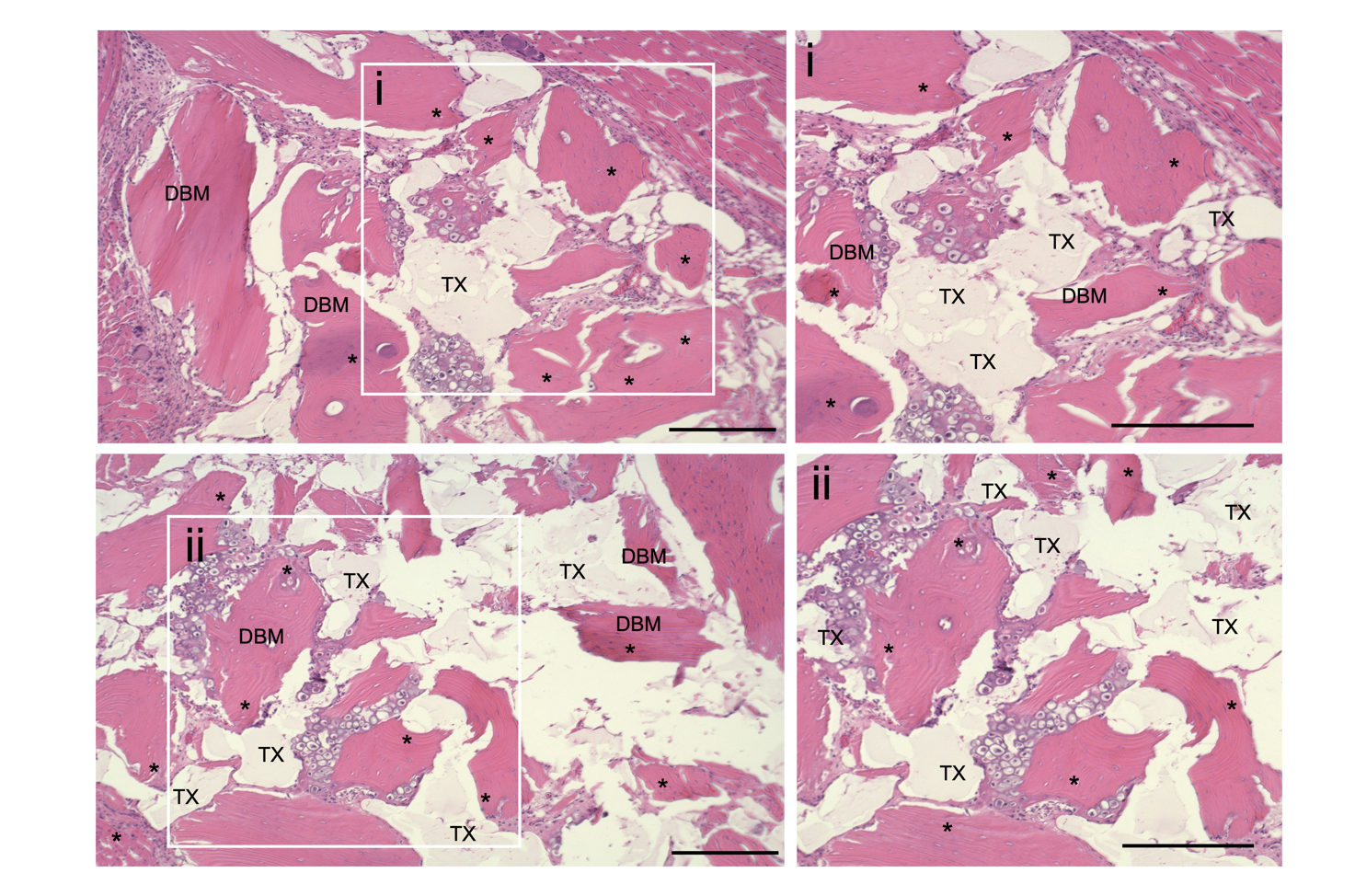


**Figure S3.** **H&E Stained of implanted sites with TX140/DBM70, 4 weeks post-implantation in hindlimbs of nude mouse models.** TX: hydrogel remnants; newly generated ectopic bone is identified with *. Scale bars are at 100 µm.


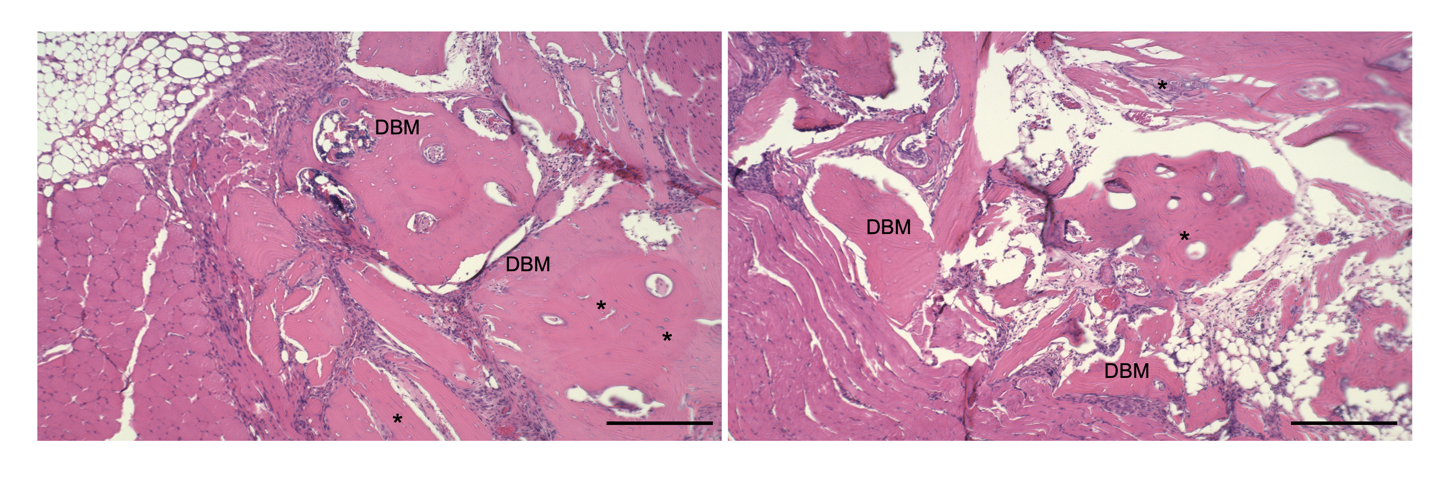
**Figure S4.** **H&E Stained of implanted sites with DBM100, 4 weeks post-implantation in hindlimbs of nude mouse models.** Newly generated ectopic bone is identified with * Scale bars are at 100 µm.

# Rabbit femoral critical size defect study

## Histology protocol

Following micro-CT the distal femurs were fixed in 10% phosphate-buffered formalin at room temperature with gentle rotation on a Labtech rotating shaker, for a minimum of 96 hours prior to gross sectioning into paraffin cassettes. The cut sections (~3 mm in thickness) were placed into embedding blocks for paraffin processing from medial to lateral in the sagittal plane (Figure S5). Each paraffin block was sectioned (5 microns) using a Leica Microtome and placed on slides for haematoxylin and eosin (H&E).

**Figure S5.** Schematic illustrating how each defect was examined at 3 levels for micro-computed tomography as well as histology sectioning to provide a robust examination. The blue rectangular geometries were the middle part of the defect and where histological evaluations were performed.

Stained sections were examined under light microscopy using an Olympus Microscope (Olympus, Japan) with a DP72 high resolution video camera (Olympus, Japan) in a blinded fashion by an experienced examiner. For histological assessment of cortical bridging, the region of interest (ROI) was defined to include the most superficial 3mm of cortical osteotomy site (*i.e.*, the most superficial paraffin block) and the tissues immediately proximal to the most superficial 3mm of the cortical osteotomy site. Histology was qualitatively assessed at each time point. The stained slides were reviewed under low magnification to provide an overview of the section for documentation purposes using 1.25x objective (scale bar = 1 mm). The sections were carefully examined at higher magnification (4x objective, scale bar = 200 microns) as well as under high power fields (10x objective, scale bare = 100 microns, 20x objective, scale bar = 50 microns, 40x objective, scale bar = 20 microns). Histological analysis was performed by a single-blinded experienced examiner for assessment of giant cells, plasma cells, lymphocytes, osteoid, woven bone, trabecular bone, osteogenic cells, and residual DBM particles.

# Extraction socket trabecular bone regeneration randomized clinical trial

## Aims of the study

In order to test the universality of the TX carrier system, we chose to combine the TX carrier system with particulate DBBM in a controlled clinical trial to assess the trabecular regenerative effects of the TX/DBBM composite. The product (TX/DBBM) and the control (DBBM-C) were placed within human extraction sockets in a tooth extraction surgical procedure, which has been commonly described as alveolar ridge preservation (ARP).^[1,2]^ The aim in APR was to provide favorable peri-implant bone volume for dental implant placement, which commonly occurs 3-6 months following ARP.^[2]^ The hypothesis of this randomized, single-blinded controlled trial was that TX combined with DBBM would have a favorable safety profile, similar handling, maintain similar bone volume, and show similar trabecular bone healing histology when compared to a commonly employed xenograft ARP procedure involving 90% DBBM combined with a 10% collagen matrix (DBBM-C) (Geistlich Bio-Oss® Collagen; Geistlich Pharma AG).^[1,2]^

## Inclusion and exclusion criteria

Ethics approval for this clinical study was granted by St. Vincent’s Hospital, Melbourne, Australia Ethics Committee (ref HREC 043/19, project ID: 52231) prior to patient enrolment. The trial protocol was registered prospectively with the Australia and New Zealand Clinical Trials Registry (ANZCTR): 12619001124123. Written and verbal informed consent was provided to all subjects. The reporting of the study follows the CONSORT (2010) guidelines.^[3]^ The study was performed in a private practice setting by a single experienced periodontist (DC) with over 20 years of experience in extraction and dental implant procedures. Patients were enrolled who needed extractions and delayed implant placement at maxillary central incisor or maxillary lateral incisor sites. The following inclusion criteria were used in this study:

- Patients > 18 years of age.
- Non-pregnant women.
- Maxillary central or lateral incisors requiring implant-supported restorations.
- Implant-supported restorations are planned for the replacement of teeth.
- Absence of acute or chronic infection at the extraction site or adjacent teeth.
- Periodontal probing pocket depths < 4mm.
- Intact facial bone wall determined by pre-extraction cone-beam computed tomographic (CBCT) examination (OP300 Maxio, 90kV, 23.4mAsand voxel size of 0.085mm).
- Confirmation of intact socket wall immediately following tooth extraction.
- Patients were excluded if they smoked cigarettes, had previous radiation therapy to the jaws, immunosuppressive therapy, active cancer, or taking drugs known to alter bone metabolism such as denosumab or bisphosphonates.

Where required, non-surgical periodontal therapy and oral hygiene instruction were provided before enrollment. All patients were required to have a full mouth plaque score (FMPS) < 25% and a full mouth bleeding score (FMBS) < 25%. The study was designed as a two-stage clinical investigation. Stage 1 was performed by a single operator (DC) as an open-label safety run-in investigation in which 3 participants were treated with the TX combined with DBBM (small particle, cancellous, 0.25 mm-1 mm, Bio-Oss^®^, Geistlich Pharma AG) (TX/DBBM). Stage 2 was performed by the same single operator and designed as a prospective multi-center, single-blinded, randomized, comparator-controlled, two (2) arm investigation where the participants were randomly distributed into two treatment groups: experimental group (TX/DBBM) or control group (DBBM-C). Patient recruitment was completed in a blinded manner to eliminate enrollment bias by the principal investigator and the treating clinician.

## Patient demographics

Fourteen patients were initially screened for participation in the study. Of the fourteen patients, 3 were subsequently excluded, one patient because of infection at the intended extraction site prior to the placement of either TX/DBBM or DBBM-C. The other 2 patients elected to withdraw from the study after enrollment due to a concern over the additional time and travel commitments required for the study. Over a 22-month recruitment period, a total of 11 consecutive patients who fulfilled the inclusion criteria were enrolled and treated in the study (summary of patient demographics Table S2). Of the 11 patients, there were 3 females and 8 males. There were 3 open-label subjects (TX/DBBM) and 8 randomized subjects (5 test and 3 control patients). A total of 11 teeth were extracted, consisting of 3 maxillary lateral incisors and 8 maxillary central incisors. All patients treated with TX/DBBM or DBBM-C returned for their follow-up visits for each time point post-operation (1 week, 2 weeks, 4 weeks, 8 weeks, and 16 weeks).

**Table S2.** Demographic Data, Group Allocation and Site Distribution of Patients ARP RCT

|  | Study Group | | | |
| --- | --- | --- | --- | --- |
|  | | *Open Label TX/DBBM* | *Experimental*  *TX/DBBM* | *Control*  *DBBM-C* |
| No. patients | | 3 | 5 | 3 |
| No. teeth | | 3 | 5 | 3 |
| Mean age (y) | | 57 | 53 | 31 |
| (range) | | 25-79 | 37-78 | 20-50 |
| Patient gender (M/F) | | 1/2 | 4/1 | 3/0 |
| Tooth type | |  | | |
| Maxillary lateral incisors | | 1 | 1 | 1 |
| Maxillary central incisors | | 2 | 4 | 2 |

## Clinical procedures

Oral Paracetamol (1g) and oral antibiotics (Amoxycillin 1g or Cephazolin 2g or Clindamycin 300mg if known penicillin anaphylaxis) were administered 30 mins prior to tooth extraction. Local anaesthesia was provided at the surgical site (2% lignocaine hydrochloride; and 1:80K adrenalin). A flapless tooth extraction technique to minimize surgical trauma was employed. Using a No. 15C surgical blade, circumferential intrasulcular supracrestal fiberotomies were performed to separate periodontal attachment prior to the initial mobilization of teeth. The teeth were then removed using fine-tipped forceps with slow rotational forces applied to the cervical area and avoiding contact with the facial alveolar bone. The post-extraction sockets were carefully debrided with curettes to remove any soft tissue tags, however, there was no definitive attempt to remove any residual periodontal ligament fibers.

Alveolar ridge preservation was then performed. For the TX/DBBM group, the TX carrier was mixed chairside with DBBM for 2 minutes prior to implantation. The resulting composite was manipulated, such that the final composite involved ~90 wt% DBBM and 10 wt% TX. The resulting TX/DBBM composite was then gently packed into the base of the socket to the level of the alveolar bone crest. For the control group, the DBBM-C porous block was hydrated with isotonic sterile saline, cut into fragments, and directly packed into the socket to the level of the alveolar bone crest. For all participants, the socket entrance was closed with a resorbable type I/III porcine collagen matrix (CM) (Geistlich Mucograft® Seal; Geistlich Pharma AG) that was sutured into position using interrupted 5-0 polyglactin sutures (Novosyn ® Quick, B/Braun, Surgical, SA. Rubi Spain). Post-surgical oral antibiotics were prescribed, Amoxicillin 500mg tds (6 days) or Cephelaxin 500mg tds (6 days) or Clindamycin 150 qid (6 days) if there was a known anaphylactic reaction to penicillin. Paracetamol 500 mg tds or Paracetamol 500 mg combined with Codeine Phosphate 30 mg was provided for inflammation and pain (if required).

Both groups were instructed not to wear removable prostheses for 7 days post-surgery. For those patients who wished to wear a removable partial denture or vacuum-formed removable prosthesis following this 7-day period, care was taken to ensure that the prosthesis did not contact soft tissues at all post-operative time points. Patients were also instructed to use an alcohol-free 0.2% chlorhexidine mouth rinse (Savacol™, Colgate^®^) diluted with 50% water daily commencing 24 hours after surgery for 7 days and to avoid brushing teeth immediately adjacent to the surgical site for 2 days followed by brushing these teeth with a manual extra soft toothbrush for 2 weeks. For those teeth not approximating the area of surgical intervention, conventional tooth brushing with a soft head (electric or manual tooth brushing) was to commence immediately post-surgically according to previous oral hygiene instructions. Post-operative follow-up meetings were scheduled 1, 2, 4, 8, and 16 weeks post-operation. Sutures were removed 1 week after surgery.

Implant surgery was scheduled for 16 weeks following tooth extraction and ARP. Following the administration of local anesthesia (2% lignocaine hydrochloride; 1:80 000 adrenalin), a full-thickness papilla sparing incision with mesial and distal divergent vertical releasing incisions was performed. Implant osteotomies were initially undertaken using a 2mm internal diameter trephine drill (Treph-2, Salvin® Dental Specialties, Charlotte NC). Implant placement was performed using 3.3mm or 4.1mm diameter tapered implants after the preparation of a final osteotomy according to the implant manufacturer’s recommendations (3.3mm BLT, 4.1mm BLT, Roxolid^®^, SLActive ^®^, Institut Straumann AG). Implants were placed in ideal 3D positions to optimize soft tissue aesthetic outcomes as has been described for anterior single tooth implant sites^[4]^ (refer to Figure S7 Supporting Information). The trephine drill including the bony core was then carefully placed in a plastic vial containing 10% neutral buffered formalin. The fixed specimen was sent to an independent pathology laboratory for hematoxylin and eosin (H&E) staining, Movat’s pentachrome staining, and histological analyses. Where required, additional grafting was undertaken prior to wound closure to improve facial soft tissue aesthetics, using a resorbable native bilayer collagen barrier membrane (NBCM) (Geistlich Bio-Guide®; Geistlich Pharma AG) and particulate DBBM. Transmucosal healing abutments were then placed or the implants were fully submerged. For those implants fully submerged, a stage II surgical procedure to allow for the placement of transmucosal healing abutment was performed 3-5 months following implant placement.

## Assessment of Safety, the extent of soft tissue healing, and the rate of tissue epithelisation in this clinical trial

**Table S3.** Safety Assessment of the administrated materials in the clinical study in this work.

| Participant ID | Age | Material | Week 1  Post-administration | | | Week 2  Post-administration | | | Week 4  Post-administration | | |
| --- | --- | --- | --- | --- | --- | --- | --- | --- | --- | --- | --- |
|  |  |  | Pain | Inflammation/ Swelling | Other Abnormality | Pain | Inflammation/ Swelling | Other Abnormality | Pain | Inflammation/ Swelling | Other Abnormality |
| 1 | 71 | TX/DBBM | No | No | No | No | No | No | No | No | No |
| 2 | 83 | TX/DBBM | No | *Yes* | No | No | No | No | No | No | No |
| 3 | 29 | TX/DBBM | No | No | No | No | No | No | No | No | No |
| 4 | 47 | TX/DBBM | *Yes, Mild* | No | No | No | No | No | No | No | No |
| 5 | 22 | DBBM-C | *Yes, Mild* | *Yes* | No | No | *Yes* | No | No | No | No |
| 6 | 60 | TX/DBBM | No | No | No | No | No | No | No | No | No |
| 7 | 26 | DBBM-C | *Yes, Mild* | *Yes* | No | No | No | No | No | No | No |
| 8 | 52 | DBBM-C | No | No | No | No | No | No | No | No | No |
| 9 | 80 | TX/DBBM | No | No | No | No | No | No | No | No | No |
| 10 | 37 | TX/DBBM | No | No | No | No | No | No | No | No | No |
| 11 | 47 | TX/DBBM | No | *Yes* | No | No | No | No | No | No | No |
| Summary |  | TX/DBBM Group | 1/8 | 2/8 | 0/8 | 0/8 | 0/8 | 0/8 | 0/8 | 0/8 | 0/8 |
|  |  | DBBM-C | 2/3 | 2/3 | 0/3 | 0/3 | 1/3 | 0/3 | 0/3 | 0/3 | 0/3 |

## Socket Preservation Measurements

Cone beam computerized tomography (CBCT) measurements were performed using a standardized protocol at 3 time points: pre-extraction, 8 weeks post-extraction, and 16 weeks post-extraction with images assessed by 2 independent specialist oral and maxillofacial radiologists (TH) and (BK). (refer to Figure S6 and Figure S7 for CBCT methodology and analysis).

All measurements were conducted by two blinded and independent radiologists. The DICOM data were imported into a viewing software (syngo.via; Siemens Healthineers AG). The baseline and 8-weeks post-graft scans were opened on the same window with the post-graft scan allocated a green color using the CBCT Color LUT tool to differentiate from the baseline scan. The scans were then fused using the automatic registration tool. Accurate fusion was verified by the observer and the visual alignment tool was used to adjust where required. The balance of visualization of either the baseline or post-graft scans could be adjusted on a scale of 0-100%. Prior to obtaining measurements, standard anatomic planes were corrected as follows. The center (intersection of the relevant two reference planes; coronal and sagittal) of the reference planes was positioned at the center of the tooth of interest. The mesiodistal reference plane was positioned parallel to the mesial-distal contact points. The apicocoronal orientation of the reference plane was adjusted to coincide with the long axis of the tooth (Figure S6).


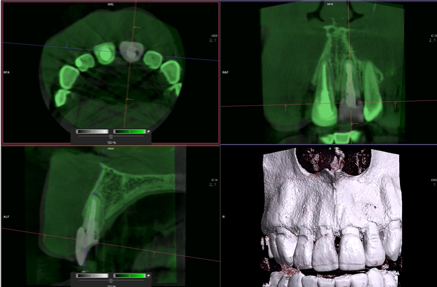


**Figure S6.** The center (intersection of the relevant two reference planes; coronal and sagittal) of the reference planes were positioned at the center of the site of interest. The mesiodistal reference plane was positioned parallel to the mesial-distal contact points. The apicocoronal orientation of the reference plane was adjusted to coincide with the long axis of the tooth.

The measurements were performed in three steps:

1. For the measurement of the baseline scan, the percentage of visualization was initially adjusted such that only the baseline scan was visible. The cross-sectional view at the site of interest was then selected. The buccopalatal measurements of the ridge perpendicular to the apicocoronal reference plane were then made using the linear measurement tool at 1mm increments from 0 to 15mm or to the nasal or maxillary sinus floor for those measurements less than 15mm (Figure S7 a,b)
2. For measurement of the 8-weeks post-graft scan, the measurements from step one were retained and the percentage of visualisation was then adjusted so that only the 8-weeks post graft scan was visible. The measurements for the post-graft scan were then performed. (Figure S7 c,d)
3. The 8-weeks post graft scan was removed and replaced with the 16-weeks post graft scan in the viewing software. Fusion of the baseline and 16-weeks post-graft scans were performed as detailed above. For measurement of the 16-weeks post-graft scan, the measurements from step two were retained and the percentage of visualisation was then adjusted so that only the 16-weeks post graft scan was visible. The measurements for the 16-weeks post-graft scan were then performed. (Figure S7 e,f).

The above measurement protocol described above ensures that the measurements performed on the baseline and the post-graft scans were at exactly the same location and plane. Measurements were made in bulk to minimize day-to-day variation of measurements by the assessors.

**Figure S7.** **Width loss measurements after grafting with TX/XG.** Orofacial measurements of the ridge perpendicular to the long axis of the tooth were obtained at 1 mm increments apical to the CEJ as identified by corresponding digits on the dotted lines. (a) For the measurement of the baseline scan, the percentage of visualization was initially adjusted such that only the baseline scan was visible. The cross-sectional view at the site of interest was then selected. The buccopalatal measurements of the ridge perpendicular to the apicocoronal reference plane were then made using the linear measurement tool at 1 mm increments from 0 to 15 mm. (b) the baseline measurements with baseline and 8-weeks post graft scans were visible. (c) for measurement of the 8-weeks post-graft scan, the measurements from step one were retained, and the percentage of visualization was then adjusted so that only 8-weeks post-graft scan was visible. The measurements for the post-graft scan were then performed. (d) The post-graft measurements with both baseline and 8-weeks post-graft scans visible. (e) for measurement of the 16-weeks post-graft scan, measurements from step two were retained and the percentage of visualization was then adjusted so that only the 16-weeks post-graft scan was visible. The measurements for the 16-weeks post-graft scan were then performed. (f) the post-graft measurements with both baseline and 16-weeks post-graft scans visible.


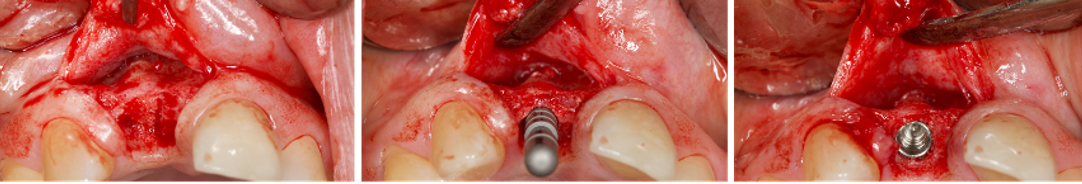


**Figure S8.** **Implant placement.** The images were taken at week 16 post-administration of TX/DBBM.

**References**

[1] I. Darby, S. T. Chen, D. Buser, *Int J Oral Maxillofac Implants* **2009**, *24*, 260.

[2] S. Bassir, M. Alhareky, B. Wangsrimongkol, Y. Jia, N. Karimbux, *Int. J. Oral Maxillofac. Implants* **2018**, *33*, 979.

[3] K. F. Schulz, *J Clin Epidemiol.* **2010**, *63*, 834.

[4] D. Buser, V. Chappuis, U. C. Belser, S. Chen, *Periodontol. 2000* **2017**, *73*, 84.
